# Biogeography of Southern Ocean Active Prokaryotic Communities Over a Large Spatial Scale

**DOI:** 10.3389/fmicb.2022.862812

**Published:** 2022-05-03

**Authors:** Claudia Maturana-Martínez, José Luis Iriarte, Sun-Yong Ha, Boyeon Lee, In-Young Ahn, Maria Vernet, Mattias Cape, Camila Fernández, Humberto E. González, Pierre E. Galand

**Affiliations:** ^1^Centro de Investigación Dinámica de Ecosistemas Marinos de Altas Latitudes (IDEAL) and Universidad Austral de Chile, Valdivia, Chile; ^2^Sorbonne Université, CNRS, Laboratoire d'Ecogéochimie des Environnements Benthiques, Banyuls-sur-Mer, France; ^3^Division of Polar Ocean Science, Korea Polar Research Institute, Incheon, Republic of Korea; ^4^Scripps Institution of Oceanography, University of California, San Diego, San Diego, CA, United States; ^5^School of Oceanography, University of Washington, Seattle, WA, United States; ^6^Sorbonne Université, CNRS, Laboratoire d'Océanographie Microbienne (LOMIC), Banyuls-sur-Mer, France; ^7^Centro COPAS Coastal, Universidad de Concepción, Concepción, Chile

**Keywords:** DNA, RNA, Southern Ocean, fjord, biogeography

## Abstract

The activity of marine microorganisms depends on community composition, yet, in some oceans, less is known about the environmental and ecological processes that structure their distribution. The objective of this study was to test the effect of geographical distance and environmental parameters on prokaryotic community structure in the Southern Ocean (SO). We described the total (16S rRNA gene) and the active fraction (16S rRNA-based) of surface microbial communities over a ~6,500 km longitudinal transect in the SO. We found that the community composition of the total fraction was different from the active fraction across the zones investigated. In addition, higher α-diversity and stronger species turnover were displayed in the active community compared to the total community. *Oceanospirillales, Alteromonadales, Rhodobacterales*, and *Flavobacteriales* dominated the composition of the bacterioplankton communities; however, there were marked differences at the order level. Temperature, salinity, silicic acid, particulate organic nitrogen, and particulate organic carbon correlated with the composition of bacterioplankton communities. A strong distance–decay pattern between closer and distant communities was observed. We hypothesize that it was related to the different oceanic fronts present in the Antarctic Circumpolar Current. Our findings contribute to a better understanding of the complex arrangement that shapes the structure of bacterioplankton communities in the SO.

## Introduction

Although microbial marine communities are the essential players of the marine ecosystem functioning, being the main drivers of the major marine biogeochemical cycles (Azam et al., 1983; Karl, 2002; Pomeroy et al., 2007), little is known about the ecological and oceanographic drivers that shape their distributions. Some known factors that affect the microbial community structure include geographical position from tropical to polar regions (Fuhrman et al., 2008; Brown et al., 2012; Ghiglione et al., 2012) and the physical-chemical parameters of the marine environment such as temperature, salinity, depth and chlorophyll-a (Chl-a) concentration (Crump et al., 2004; DeLong, 2006; Falcón et al., 2008; Campbell and Kirchman, 2013; Fortunato et al., 2013; Sunagawa et al., 2015; Herlemann et al., 2016). Furthermore, oceanographic features such as water masses, oceanic fronts, and advection have also been described as physical barriers that can limit the dispersal of microorganisms and, at the same time, influence the structure of the communities at the ocean scale (Galand et al., 2010; Agogué et al., 2011; Wilkins et al., 2013a).

The Southern Ocean (SO) plays an important role in global ocean circulation, biogeochemical cycles, and climate (Broyer and Koubbi, 2014). The surface of the SO is composed of several distinct zones defined by transitions in surface water temperatures and density (Orsi et al., 1995; Sokolov and Rintoul, 2002). The Antarctic Circumpolar Current (ACC) divides the SO into three major zones: the Subantarctic Zone (SAZ), the Polar Frontal Zone (PFZ), and the Antarctic Zone (AAZ) (Pollard et al., 2002). These three major zones are separated from each other from north to south by the Subtropical Front (STF), the Subantarctic Front (SAF), the Polar Front (PF), and the Southern Fronts (SF) (Whitworth and Nowlin, 1987; Orsi et al., 1995; Sokolov and Rintoul, 2002). The Southern Fronts (SF) belonging to the ACC are divided into (i) the southern ACC front (sACCf) and (ii) the Southern Boundary of the ACC front (sbACC) (Sokolov and Rintoul, 2002).

Oceanic fronts are regions where environmentally distinct water masses meet, creating sharp physicochemical gradients over fine spatial scales (Belkin et al., 2009). Therefore, each zone and fronts possess characteristic temperature, salinity, and productivity levels (Pollard et al., 2002; Sokolov and Rintoul, 2002) and tend to have distinctive biological communities (Sokolov and Rintoul, 2002). However, as a consequence of climate change, waters toward the pole side of the ACC waters have become warmer and more saline, while those to the north are cooler and fresher (Ning et al., 2008; Haumann et al., 2016).

The West Antarctic Peninsula (WAP) and the South Shetland Islands in the AAZ are characterized by varied and complex coastal ecosystems and oceanographic processes (Prezelin et al., 2004; Schofield et al., 2010). This coastal ecosystem is subjected to strong seasonal and interannual variability driven by global atmospheric and oceanic changes (Morley et al., 2020). The increasing atmospheric and ocean temperatures registered along the Antarctic Peninsula (AP) have contributed to the collapse of numerous ice shelves and the retreat of many glaciers (Kim and Ducklow, 2016; Morley et al., 2020). Some of the consequences of ice melting and the resulting freshwater input for the AP coastal ecosystems include salinity decrease and stratification of the water column, limited light penetration, increased iron availability, and opening of new areas for primary production (Schofield et al., 2010; Höfer et al., 2019; Hopwood et al., 2020; Morley et al., 2020). More specifically, along King George Island, many semi-enclosed embayments such as Maxwell Bay (MB) and its tributary Marian Cove (MC) are exposed to freshwater input from glaciers surrounding the bay and oceanic water exchange with the Bransfield Strait (BS) (Llanillo et al., 2019). The evidence indicates that this area is experiencing accelerated warming due to the retreat of glaciers such as the glacier associated with MC, which has retreated 1.7 km from 1956 to 2012 (Ahn et al., 2016). As a consequence of the retreat of the MC glacier, the structure and function of the megabenthic epifauna have been affected and benthic diatom blooms have been recorded as unusual in this area (Moon et al., 2015; Ahn et al., 2016).

Several studies of diversity and community structure on bacterioplankton at regional scale have been carried out in the SO (Piquet et al., 2011; Signori et al., 2014; Hernández et al., 2015; Luria et al., 2016; Dinasquet et al., 2017; Picazo et al., 2019; Alcamán-Arias et al., 2021). However, only a few studies have reported results from a large-scale investigation (Wilkins et al., 2013a,b; Luria et al., 2016), and none has targeted the active fraction of the microbial communities. The seasonal dynamics of some heterotrophic prokaryotic communities have, for instance, been described in association with phytoplanktonic blooms in nutrient-enriched areas of the SO (Obernosterer et al., 2011; Kim and Ducklow, 2016; Luria et al., 2016; Liu et al., 2019, 2020). In open oceanic areas, physical parameters such as temperature, depth, ice melting, advection, oceanic fronts, and water circulation have been described as the major drivers shaping prokaryotic community structure (Piquet et al., 2011; Wilkins et al., 2013a,b; Alcamán-Arias et al., 2021). In some coastal areas of the Antarctic region such as bays and fjords, the combination of environmental parameters, including salinity, nutrients, and dissolved/particulate organic/inorganic matter, are described as major structuring factors (Moreno-Pino et al., 2016; Picazo et al., 2019; Kim et al., 2020). While the composition of the bacterioplankton community in the SO shows some variability across the different areas, members of the classes *Gammaproteobacteria, Alphaproteobacteria*, and *Bacteroidetes* tend to dominate the number of sequences (Abell and Bowman, 2005; Ghiglione et al., 2012; Wilkins et al., 2013b; Baltar et al., 2016; Liu et al., 2019; Logares et al., 2020). Among *Alphaproteobacteria*, SAR11 often dominates in both coastal and open ocean areas (López-García et al., 2001; Murray and Grzymski, 2007; Giebel et al., 2009; Piquet et al., 2011; Ghiglione et al., 2012). The clade is more abundant in Subantarctic and PF areas where high nitrogen and low chlorophyll conditions prevail, giving it a competitive advantage compared to the Antarctic zone where phytoplankton blooms result in increased concentrations of high molecular weight dissolved organic matter that has been found to decrease their abundance (Giebel et al., 2009; Ghiglione and Murray, 2012). For *Gammaproteobacteria*, the order *Alteromonadales* includes heterotrophs with broad substrate preferences (Bowman et al., 1997). Within this order, *Cowellia* genera is a common member of polar systems with adaptation to cold environments through the production of cold active extracellular enzymes (Methe et al., 2005). Among *Bacteroidetes*, members of the class *Flavobacteria* are the major components of the planktonic communities in the SO, especially prevalent in particle attached communities and in association with phytoplankton blooms (Abell and Bowman, 2005). This class has a well-described ability to degrade organic matter, suggesting an important role in the remineralization of primary production products (Kirchman, 2002). The predicted climate changes in different areas that comprise the SO, such as water temperature increase, ocean acidification, changes in salinity, and water stoichiometry among others, are expected to influence the composition of the microbial communities in terms of abundance and distribution (Pearce, 2008), demanding a better understanding of the key patterns of microbial ecology and biogeography as well as the specific ecosystem of the SO.

The objective of this study was to test how geographical distance and environmental parameters shape prokaryotic community composition in the SO. We explored the distribution, abundance, and diversity of prokaryotic communities across different oceanographic areas in the surface layer of the SO. We then analyzed both the total and active fraction of the communities, based on the 16S rRNA gene and 16S rRNA-based, using samples collected over a spatial transect spanning over 6,500 km of the SO and the WAP continental shelf, terminating in a coastal fjord system on King George Island. Our study traverses large regions of the ocean but also includes small spatial gradients (fjords), which should be able to identify the impact of both physical (e.g., geographical distance, oceanic fronts) and environmental barriers in the dispersal and structuring of bacterioplankton communities in the SO.

## Materials and Methods

### Study Area and Sampling

**Figure 1:**
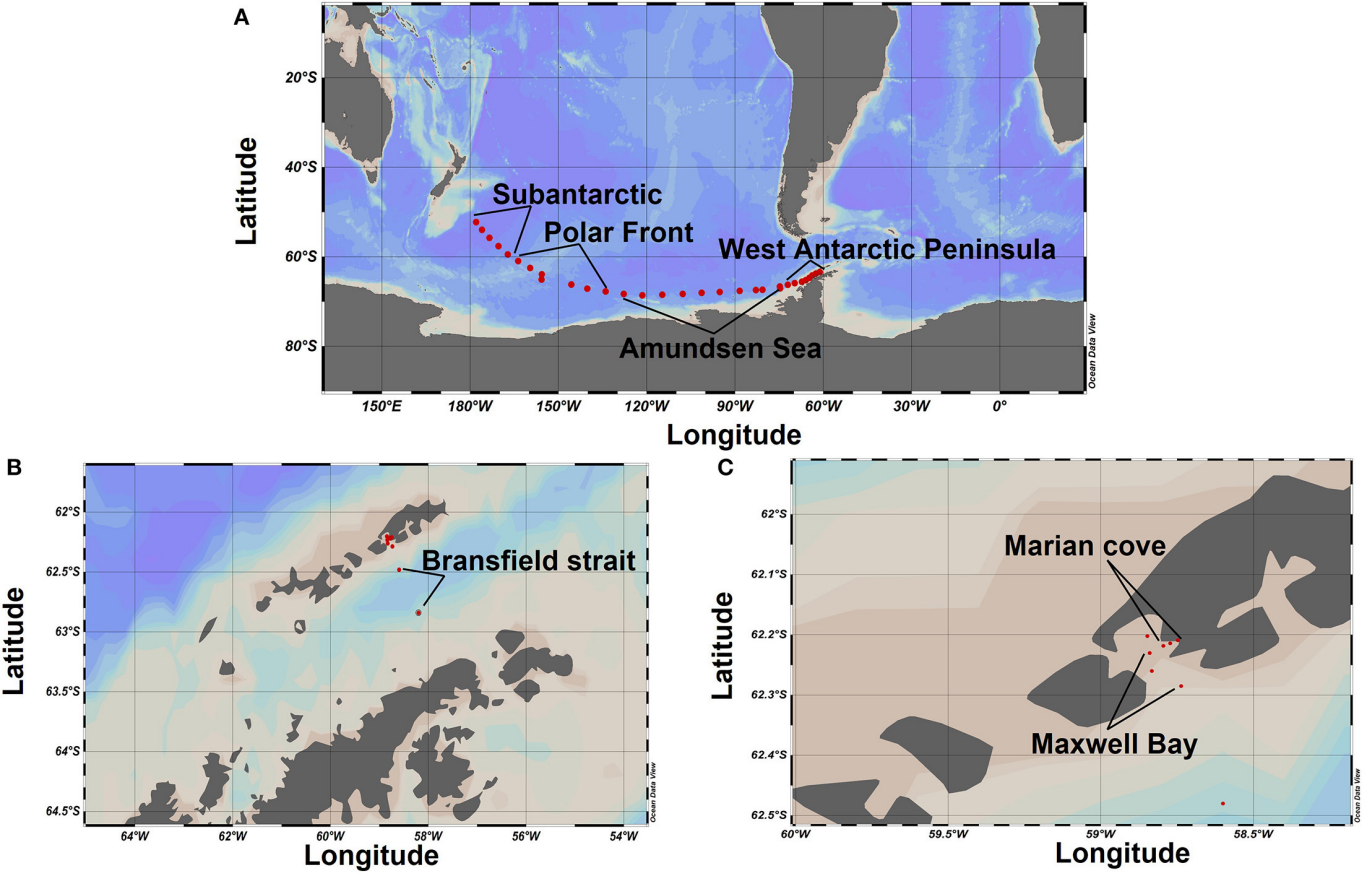
Study areas and sampling sites. **(A)** Transect Christchurch, New Zealand, to the West Antarctic Peninsula. **(B)** Bransfield Strait. **(C)** Transect from Maxwell Bay to Marian cove fjord.

The field campaign was conducted from March to May 2018 aboard the icebreaker RV ARAON as a part of the expedition ANA08D of the Korean Polar Research Institute (KOPRI). Samples were taken along a transect from Christchurch New Zealand to the WAP (SO, Figure 1A), through the BS into MC fjord (Figures 1B,C) crossing the different oceanic fronts of the SO (Supplementary Figure 1). For the SO transect, a total of 31 stations were sampled (Figure 1A). The first 20 stations were sampled every 12 h, whereas the rest of the stations (from 21 to 31) were sampled every 6 h. The water was collected directly from the ship's clean seawater flow-through system, with intake located 7 m below the ocean surface. For the BS - MC transect, a total of 8 stations were sampled: 2 stations in the BS (Figure 1B), 3 stations in MB, and 3 stations in MC Fjord (Figure 1C). Seawater from four different depths (0, 10, 25, and 50 m) was collected at each station from a rosette system with Niskin bottles. The 39 stations sampled on the SO transect were grouped into 7 zones according to their geographic location and oceanographic parameters and oceanographic parameters (water masses, major oceanographic fronts, and coastal vs. oceanic influence). The first 5 stations were named as Subantarctic zone (SA) (Figure 1A). The subsequent 5 stations were grouped as Polar Front (PF) zone, followed by 10 stations that were grouped as Amundsen Sea (AS) zone (Figure 1A). The next 11 stations were grouped as West Antarctic Peninsula (WAP) (Figure 1A) and the 2 stations sampled on the Bransfield Strait (BS) remain under the same name (Figure 1B). Finally, the last 6 stations were divided into Maxwell Bay (MB) zone and Marian Cove (MC) zone (Figure 1C) (geographical location of each zone is provided in Supplementary Material).

For microbial biodiversity analysis, 2 L of water was filtered sequentially onto the 3.0 and 0.22-μm pore size polycarbonate membrane filters (MilliporeSigma, Massachusetts, USA) and stored in RNAlater (Thermo Fisher Scientific, Massachusetts, USA) at −80°C (liquid nitrogen) until analysis. Hydrographical data including salinity, photosynthetically active radiation (PAR), and temperature were recorded using a Sea-Bird SBE 9 CTD (Sea Bird Scientific, USA).

Samples for quantification of Chl-a and dissolved inorganic nutrients were collected from 500 ml of seawater filtered through the 0.7-μm glass fiber filters (Whatman GF/F, MilliporeSigma, Massachusetts, USA). For total Chl-a determination, 100–250 ml of seawater was filtered through GF/F Whatman glass fiber filters, 0.7 μm nominal pore size (Whatman ®, Maidstone, UK), immediately frozen at −80°C and stored overnight. Filters were later extracted in 90% acetone at −20°C and analyzed *via* fluorometry (Turner Design TD-700, California, USA) at Scripps Institution of Oceanography using standard protocols (Holm-Hansen et al., 1965). Samples were calibrated using pure Chl-a (Sigma Aldrich^®^, Misuri, USA) in 90% acetone (ACS grade), with concentration measured spectrophotometrically (Jeffrey and Humphrey, 1975). For microbial abundances, two separated samples (1,350 ml) were taken in 2-ml cryovials, fixed with glutaraldehyde (0.1% final concentration), and stored in darkness at −80°C until laboratory analysis at the Laboratory for Oceanographic Processes and Climate (Universidad de Concepción, Chile) by flow cytometry method (Marie et al., 2000). To characterize dissolved organic carbon (DOC), seawater samples were taken in duplicate and filtered through the 0.22-μm pore size filters (Whatman ® Nucleopore^TM^, Maidstone, UK) into pre-combusted (450°C) glass flask and acidified with hydrochloric acid at 37%. DOC samples were analyzed with high-temperature oxidation (HTCO) method (Sugimura and Suzuki, 1988) using a TOC-V system (Shimadzu, Kyoto, Japan). To maintain quality control of the sample analyzed, a 5-point calibration curve of seawater DOC reference standards was made. In addition, seawater DOC reference standards produced by the Hansell CRM program (http://www.rsmas.miami.edu/groups/organic-biogeochem/crm.html) were also analyzed each day. To maintain the highest quality data control, samples were systematically checked against low-carbon water and deep and surface reference waters every sixth analysis (Hansell and Carlson, 1998). The between-day precision in the DOC measurement was 1–2 μm, or a CV of 2–3%. Dissolved nutrient samples (phosphate, silicate, nitrite + nitrate, nitrogen, and ammonium) were analyzed onboard ARAON vessel using a 4-channel continuous Auto-Analyzer (QuAAtro, SEAL Analytical, Southampton, UK) according to the manufacturer's instruction and standard colorimetric methods (Parsons, 2013). The channel configurations and reagents were prepared according to the “QuAAtro Applications.” Standard curves were run with each batch of samples using freshly prepared standards that spanned the range of concentrations in the samples. Water samples for particulate organic carbon (POC) and particulate organic nitrogen (PON) were filtered through the 25-mm GF/F filters (precombusted 450°C, 4 h) (Whatman ®, Maidstone, UK). POC samples were stored frozen at −20°C until analysis. To remove the dissolved inorganic carbon, the filters were fumed with HCl overnight before analysis. Total amount of POC or PON was measured using an Elemental Analyzer at the stable isotope laboratory at the University of Hanyang, Korea.

### DNA and RNA Extraction and Sequencing

For nucleic acid extraction, the 0.2-μm frozen filters were cut with sterilized scissors into small pieces and incubated for 45 min at 37° in 840 μl of lysis buffer (40 mmol L-1 EDTA, 50 mmol L-1 Tris hydrochloride pH 8.3, and 0.75 mmol L-1 sucrose) with 50 μl of lysozyme solution (20 mg ml-1) (Thermo Fisher Scientific, Massachusetts, USA), followed by a second incubation with 50 μl of 20% sodium dodecyl sulfate (SDS) (Thermo Fisher Scientific, Massachusetts, USA) and 10 μl of proteinase K (20 mg ml^−1^) (Thermo Fisher Scientific, Massachusetts, USA). Extraction of DNA and RNA was then performed simultaneously from the lysate using an AllPrep DNA/RNA kit (Qiagen Inc, Germantown, USA) following the manufacturer's instructions. Purified RNA was subjected to a DNAse treatment with a Turbo DNAse kit (Invitrogen^TM^ Ambion^TM^ Thermo Fisher Scientific, Massachusetts, USA) to remove residual traces of genomic DNA (gDNA). The quality and the quantity of the extracted DNA and RNA were measured by spectrophotometry NanoDrop 2000 (Thermo Fisher Scientific, Massachusetts, USA). The RNA samples were reverse-transcribed to cDNA with random primers using the SuperScript^TM^ VILO^TM^ cDNA synthesis kit (Invitrogen^TM^, Thermo Fisher Scientific, Massachusetts, USA) following the manufacturer's protocol. For both the DNA and cDNA, the V4-V5 region of the 16S rRNA gene was amplified using universal primers 515FB- GTGYCAGCMGCCGCGGTAA and 926R- CCGYCAATTYMTTTRAGTTT (Parada et al., 2016). Amplification and sequencing on Illumina Miseq (Illumina®, California, USA) were conducted in the commercial laboratory Integrated Microbiome Resource (IMR, Halifax, Canada) according to the protocol published earlier (Comeau et al., 2017).

### Sequences Analyses

All the reads that had a mismatch with the 16S rRNA gene primers contained ambiguous nucleotides (*N*) or were <300 bp long beyond the forward primer were removed. In addition, stringent quality trimming criterion were applied to remove reads that had ≥10% of bases with Phred values <27. This procedure is recommended to ensure that when clustering at 97% or more, the influence of erroneous reads is minimized (Huse et al., 2010; Kunin et al., 2010). The sequences were then de-replicated and clustered at a 99% sequence similarity threshold using UCLUST (Edgar, 2010) for *de novo* OTU picking. Representative sequences were classified against the SILVA v.128 database (Quast et al., 2013). Sequence data analyses were conducted with Pyrotagger pipeline (Kunin and Hugenholtz, 2010). Sequences selected for further analysis were compared manually to the GenBank database by BLAST. Putative chimeric sequences were removed. They were identified as sequences having the best BLAST alignment <90% of the trimmed read length to the reference database and >90% sequence identity to the best BLAST match.

### Statistical Analyses

The Shannon index was calculated to compare community diversity. The OTU sequence abundance table was transformed with an Hellinger transformation (Legendre and Gallagher, 2001), and an non-metric multidimensional scaling (NMDS) based on the Bray–Curtis dissimilarity was conducted to visualize similarities in community composition between samples with the Vegan package. These calculations were done with the Vegan package (Oksanen et al., 2019) in R 3.5.3 (R Core Team, 2018). Significant differences in community structure among the different variables were tested with permutational multivariate analysis of variance (PERMANOVA) with the adonis function. Indicator species analysis was conducted using the multipatt function of the indicspecies package in R (De Cáceres et al., 2012). To explore the impact of the environmental variables on the OTU data set and control the possible effect of geographical distance, a partial canonical correspondence analysis (CCA) was computed with the function “cca” from the Vegan package. Distance–decay relationship between community assemblages was quantified using a linear model (function lm), based on the pairwise Bray–Curtis dissimilarity and the geographical distance separating each pair of communities. The geosphere package (Hijmans et al., 2017) was used to calculate the geographical distance among communities with the function distHaversine.

## Results

### Environmental Parameters

**Figure 2:**
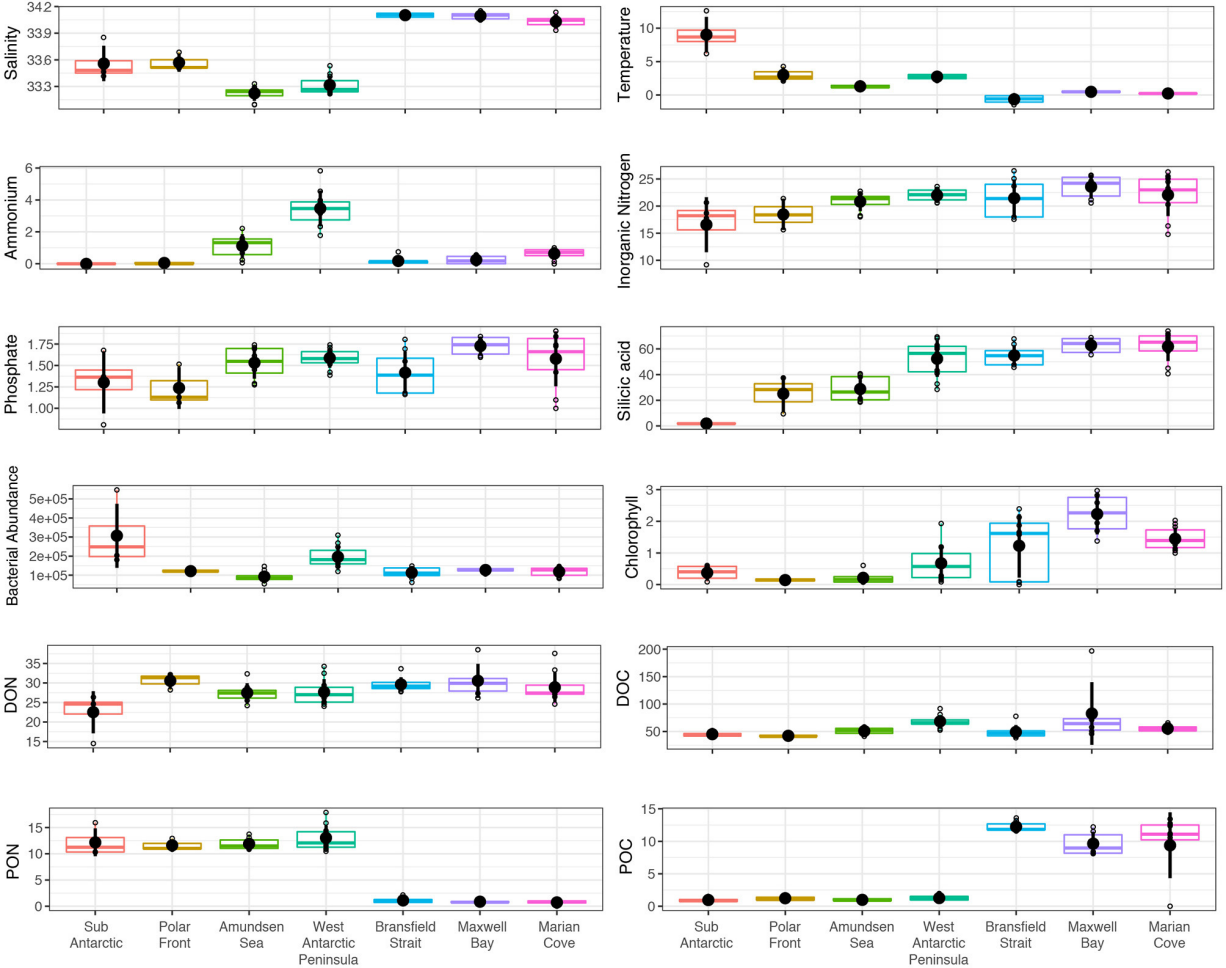
Environmental variables across the seven zones. Variables are ordered from Subantarctic to Marian Cove fjord. For the Bransfield Strait, Maxwell Bay and Marian Cove zones an average of the values corresponding to each variable was used due to multiple depths sampled (0, 10, 25, and 50 m). Variables are as follows: temperature (°C), ammonium (μm), inorganic nitrogen (NO_3_ + NO_2_) (μm), phosphate (μm), silicic acid (μm), bacterial abundance (cells/ml), chlorophyll-a (μg L^−1^), dissolved organic nitrogen (μm), dissolved organic carbon (μm), particulate organic nitrogen (μm), and particulate organic carbon (μm).

The differences in environmental conditions were observed across the seven zones through the SO transect (Figure 2). Temperature decreased from 12 to −1.4°C from SA to MC zones (−1.4°C). Chl-a concentrations varied from 0.06 to 3 μg L^−1^, with the lowest values generally found in the first 3 zones of the transect (SA, PF, and AS), and the highest values in MB and MC. Chl-a concentrations showed the highest variability in the BS zone, with values ranging from 0.06 to 2.3 μg L^−1^. Salinity showed comparatively lower variability across the zones with values ranging from 33.1 to 34.1 and maxima in salinity found in the BS, MB, and MC zones. Ammonium concentrations were generally low (<1 μm) except for the WAP zone where values fluctuated from 2.2 to 5.8 μm. For inorganic nitrogen (NO_3_+NO_2_), an increment in the concentrations was observed across the transect from SA to MC zones (13.25–22 μm, respectively) with the highest average value recorded for the MB zone (23.5 μm). Phosphate concentrations did not show strong variations across the transect (ca. 1.5 μm). A sharp increase in the concentrations of silicic acid was observed across the transect, with the lowest values for the SA zone (0.8 μm) and the highest values for the MC zone (~70 μm). An opposite pattern in concentration was noticed for PON and POC with the highest values of PON (~17–10 μm) associated with the main SO transect (SA to WAP zones), and highest POC values (~13–8 μm) associated with the final part of the transect (BS to MC). Dissolved organic nitrogen (DON) concentration values ranged from ~24 to 33 μm across the transect with the highest values measured for the PF zone. Finally, DOC concentrations varied between 45 and 80 μm across the transect.

### Overall 16S RRNA Gene and 16S RNA-Based Community Composition and Diversity

We obtained a total of 3,185,567 16S rRNA-based/16S rRNA gene sequences of Bacteria and Archaea, from 63 samples (Supplementary Table 1). A total of 95,515 different OTUs were obtained, of which 91,860 were assigned to Bacteria and 665 to Archaea.

**Figure 3:**
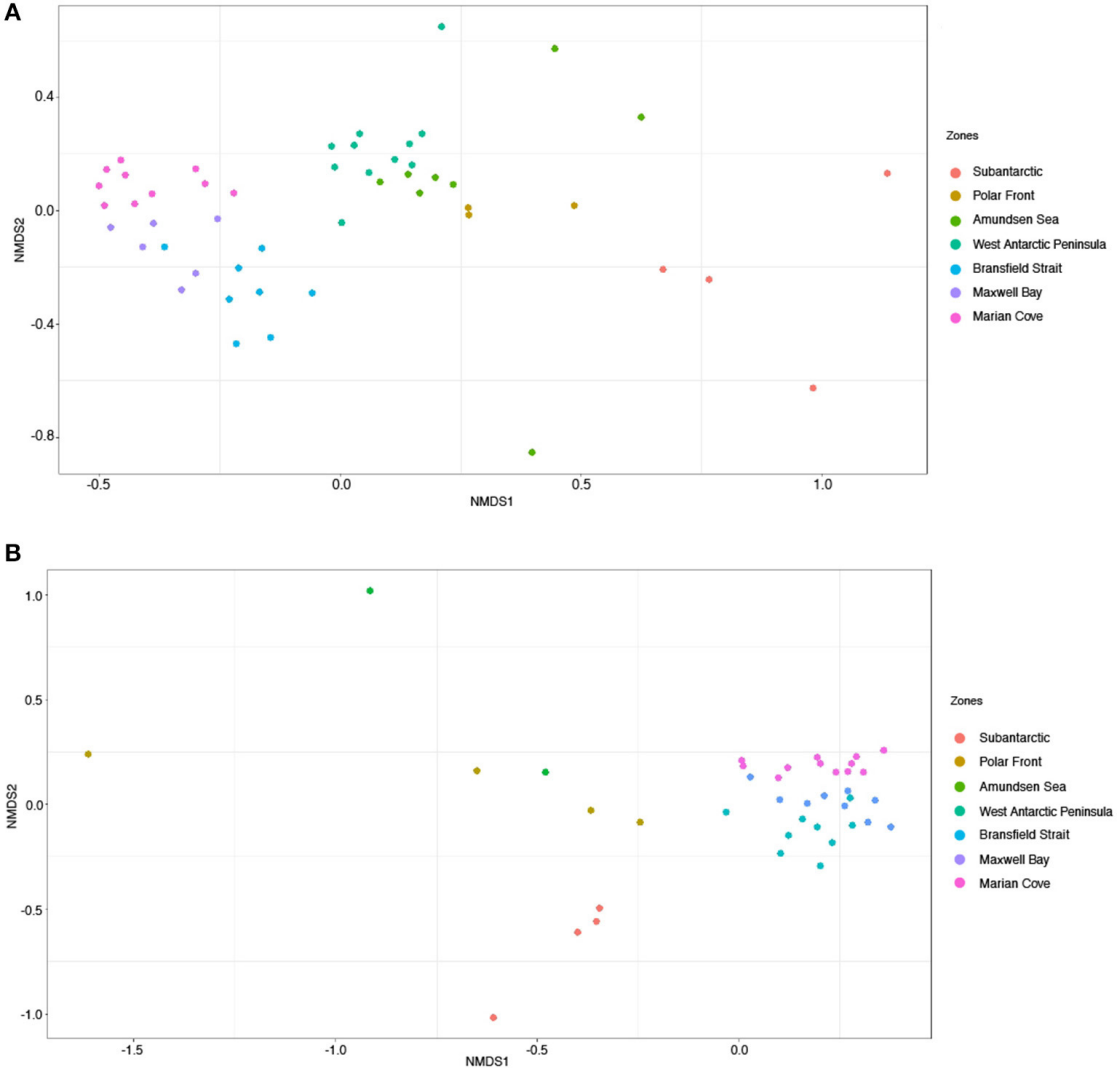
Non-metric multidimensional scaling ordination (NMDS) based on Bray–Curtis dissimilarity of microbial communities across the seven zones. Colors represent zones. **(A)** Corresponds to 16S rRNA-based fraction and **(B)** to 16S rRNA gene fraction. **(A)** Stress = 0.14, **(B)** stress= 0.13.

We compared the microbial community composition between zones based on both the active fraction of the community (16S rRNA-based) and the total fraction of the community (16S rRNA gene). The 16S rRNA-based and 16S rRNA gene fractions were separated on the NMDS ordination (PERMANOVA, *p* = 0.001) (Figure 3, Supplementary Figure 2, and Supplementary Table 2). The dissimilarity between fractions was highest in the zones AS and PF (Supplementary Figure 3). However, both fractions evidenced significant variation in the composition of the communities among zones (PERMANOVA, *p* = 0.001) (Table 1 and Supplementary Table 2).

**Table 1:** PERMANOVA testing the effects of variable zones (Subantarctic, PF, AS, AP, BS, MB, and MC) and depth (0, 10, 25, and 50 m) in each of the fractions (16S rRNA gene/ 16S rRNA-based).

**Samples**	**Fraction**	**Source of variation**	**Df**	**MS**	***F***	**R2**	***P***
All set of samples	16S rRNA gene	Zones (all)	5	0.691	3.838	0.356	**0.001**
	16S rRNA-based	Zones (all)	6	0.859	3.950	0.351	**0.001**
	16S rRNA gene	Zones (BS, MB, MC)	2	0.487	3.106	0.175	**0.001**
Costal samples with different depths		Depth (0, 10, 25, 50)	3	0.287	1.832	0.155	**0.030**
	16S rRNA-based	Zones (BS, MB, MC)	2	0.627	3.418	0.235	**0.001**
		Depth (0, 10, 25, 50)	3	0.280	1.528	0.158	0.053
	16S rRNA gene	Zones (BS, MB, MC)	2	0.348	2.153	0.234	**0.001**
Surface costal samples		Depth (0, 10)	1	0.220	1.361	0.148	**0.023**
	16S rRNA-based	Zones (BS, MB, MC)	2	0.456	2.523	0.272	**0.001**
		Depth (0, 10)	1	0.239	1.325	0.143	0.100

*Key to abbreviations and column headings: D.f, degrees of freedom; MS, mean square; F, F ratio; R^2^, coefficient of determination; P, p-value; BS, Bransfield Strait; MB, Maxwell Bay; MC, Marian Cove. Significant results are in bold*.

**Figure 4:**
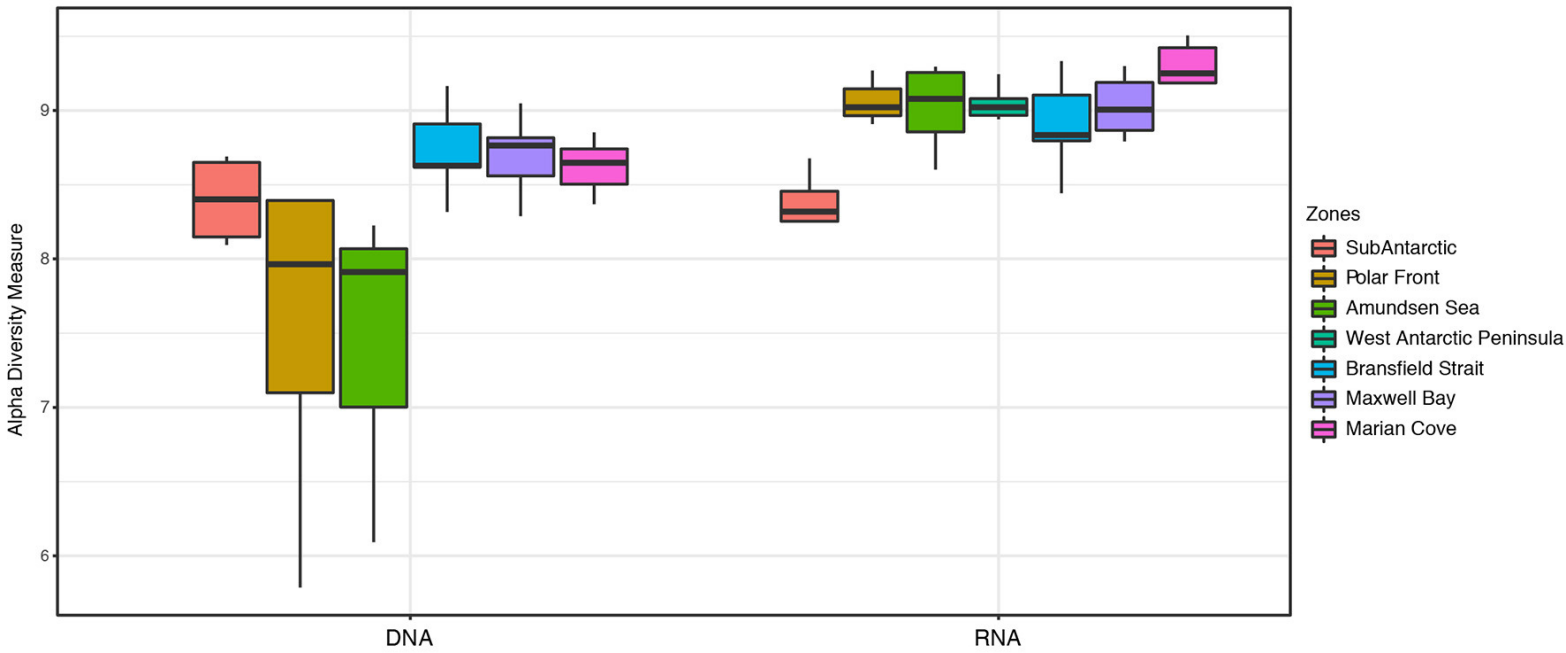
Shannon α-diversity index across seven zones, comparing 16S rRNA gene and 16S rRNA-based fractions of the microbial community from surface samples (0, 7, and 10 m).

The Shannon diversity index (α-diversity) showed a higher diversity for the 16S rRNA-based community compared to the 16S rRNA gene community in almost all the zones (Figure 4). In general, the distribution of the α-diversity in the 16S rRNA-based community was less variable (H = ~9) compared to the distribution of α-diversity observed for the 16S rRNA gene community (Figure 4). 16S rRNA gene communities in the PF and AS zones showed a high range of α-diversity values (H = 7–8.4) compare to the values of α-diversity exhibited in the 16S rRNA-based community (H = ~9) (Figure 4). The SA zone showed the lowest values of α-diversity in the 16S rRNA-based community (H = 8.3); however, these values were similar to those observed for the SA zone in the 16S rRNA gene community with a very similar median (H = 8.4) (Figure 4). The highest values of α-diversity for the entire data set were obtained in the MC zone for the 16S rRNA-based community (H = 9.2) (Figure 4).

When comparing the community composition of the coastal samples (BS, MB, and MC) with different depths (0, 10, 25, and 50 m), the variable depth was not significant at the 5% level in the 16S rRNA-based fraction (PERMANOVA, *p* = 0.053) compared to the 16S rRNA gene fraction where variation in depths was significant (PERMANOVA, *p* = 0.03) (Table 1). Additionally, in the 16S rRNA-based fraction, no significant variation in depth was found when only surface (0 and 10 m) coastal samples (BS, MB, and MC) were considered (PERMANOVA, *p* = 0.1; Table 1).

We focused the remainder of our analysis on the surface (0, 7, and 10 m) 16S rRNA-based fraction because it included data for all seven sampled zones, as opposed to the 16S rRNA gene fraction where the data for the WAP zone were missing. However, 16S rRNA gene analysis can be found in Supplementary Material.

### Environmental and Distance Effects on Active Community Composition

The partial CCA between the 16S rRNA-based community composition distance matrix and environmental variables, controlling for the effect of geographical distance, showed that 27% of the community variability was explained by environmental variables (i.e., salinity, temperature, ammonium, inorganic nitrogen, Chl-a, phosphate, silicic acid DOC, DON, POC, and PON), whereas 10% could be attributed to a geographical distance effect (Supplementary Table 3). The variables structured the samples along the first axis (CCA1) separating them into two groups, (i) main SO transect (SA, PF, AS, and WAP) and (ii) costal samples (BS-MB-MC). Communities in first group were strongly correlated with temperature (*r* = 0.79) and PON (*r* = 0.72), whereas the communities in the second group correlated strongly with silicic acid (*r* = −0.71), salinity (*r* = −0.66), and POC (*r* = 0.66) (Supplementary Figure 4 and Supplementary Table 4).

**Figure 5:**
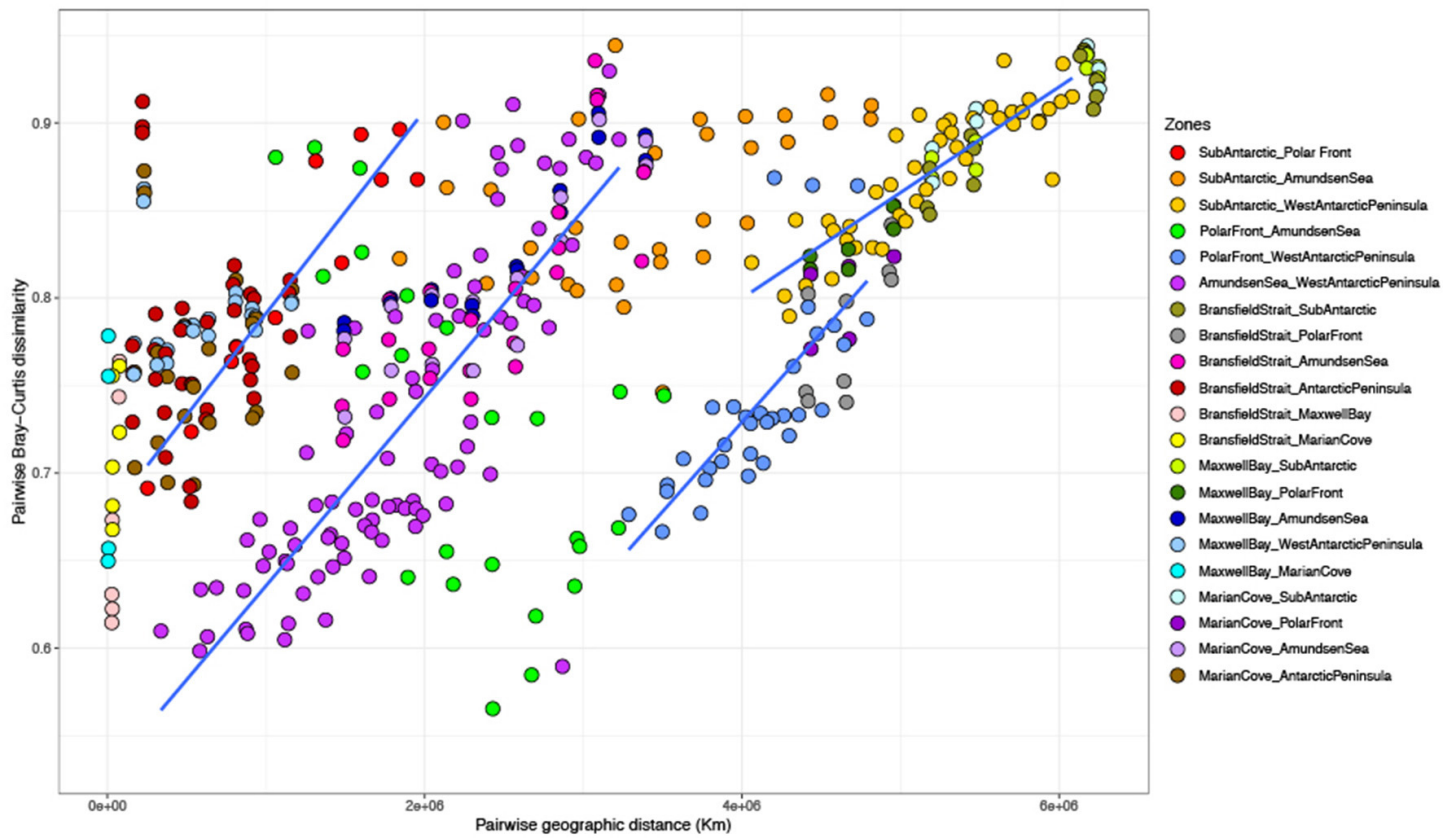
Relationship between pairwise geographical distance (km) and pairwise Bray–Curtis dissimilarity of 16S rRNA-based community composition from surface (0, 7, and 10 m) among zones.

The distance effect detected with the partial CCA was explored by comparing the geographical distance separating two samples against the dissimilarity in community composition (Bray–Curtis) (Figure 5). An overall comparison showed no significant linear relationship between geographical distance and community composition. However, when comparing specifically the different zones against each other, there was a significant correlation in 17 of the 21 comparisons (Figure 5 and Supplementary Table 5). The strongest relationships were for the more remote combinations such as SA vs. WAP (*r* = 0.76), BS vs. SA (*r* = 0.8), MB vs. SA (*r* = 0.84), MB vs. AS (*r* = 0.75), MC vs. SA (*r* = 0.88), and MC vs. AS (*r* = 0.7) (Supplementary Table 5). However, some strong and significant distance–decay patterns were also detected in closer zones such as SA vs. PF (*r* = 0.87), AS vs. WAP (*r* = 0.65), and BS vs. AS (*r* = 0.62) (Supplementary Table 5).

### Taxonomic Composition of the Active Fraction

**Figure 6:**
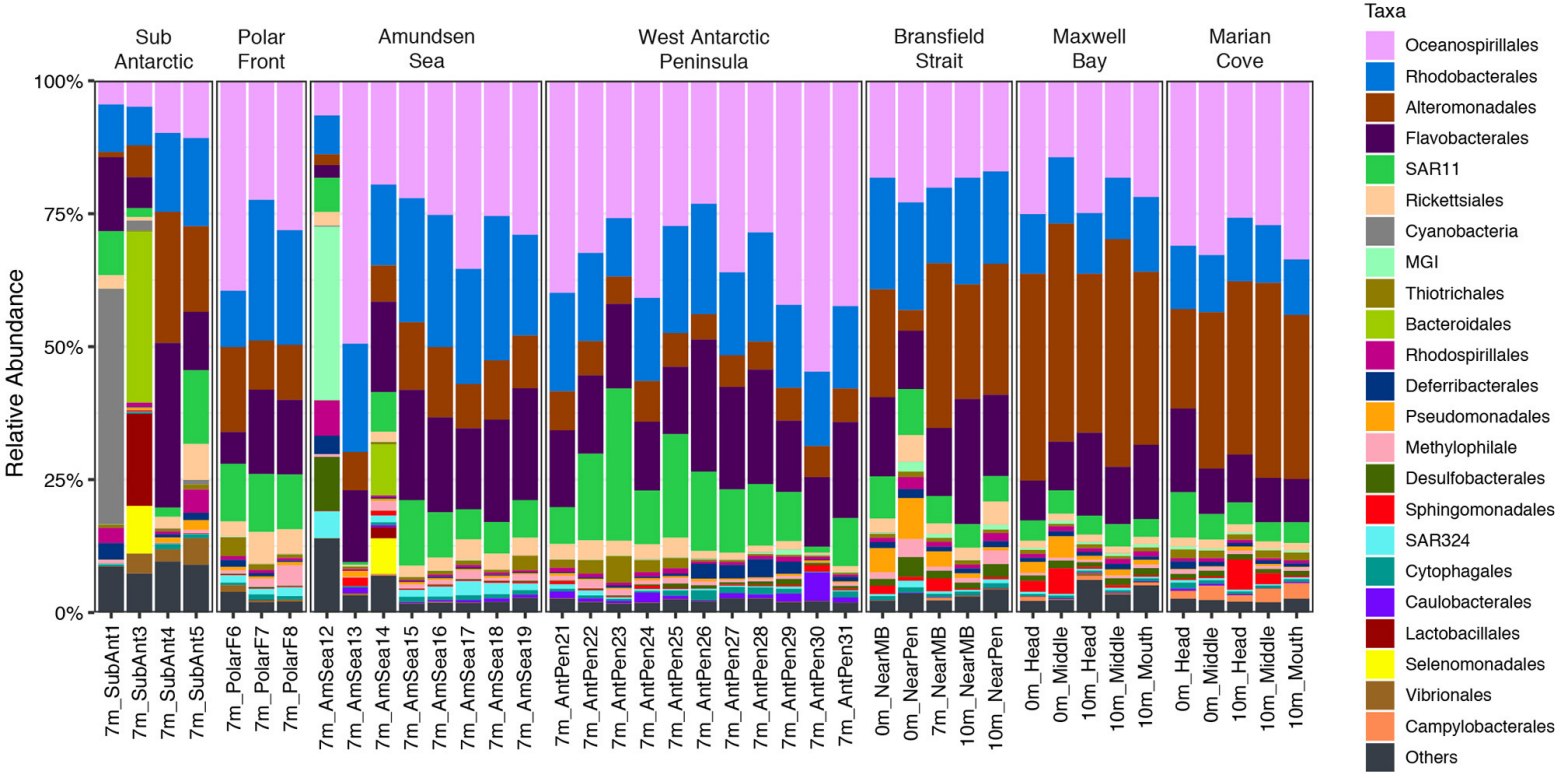
Relative abundance of taxonomical groups at the order level in the 16S rRNA-based fraction of each zone in the upper water layer (0, 7, and 10 m).

The taxonomy composition at order level showed that across the entire data set, the *Oceanospirillales* (28%), *Alteromonadales* (15.9%), *Rhodobacterales* (16%), and *Flavobacteriales* (13.9%) were the dominant orders (Figure 6). The ubiquitous SAR11 (8%) was found as a major component of the community in all the zones, showing the larger percentage on the WAP zone (12.6%).

However, distinct taxonomic compositions were also observed in each zone. The SA zone was dominated in its first two sampling points by *Cyanobacteria* (20%), *Bacteroidales* (18%), and *Lactobacillales* (9.7%) (Figure 6). The orders *Rickettsiales* (4%), *Thiotrichales* (1.6%), and *Methylophilales* (2.2%) increased in relative abundance in the PF zone (Figure 6). MGI (*Thaumarchaeota*) dominated in one of the samples of AS zone (32.6%) (Figure 6). *Desulfobacterales* (1.2%), *Rhodosporilalles* (1.1%), and SAR324 (1.9%) increased in relative abundance in the AS zone. The WAP zone was dominated by *Oceanospirillales* (35.9%), meanwhile, in the same zone, *Alteromonadales* (6.1%) exhibited the lowest values found across the entire transect.

The last 3 zones, BS, MB, and MC, showed a similar taxonomic composition dominated by *Alteromonadales* (29.2%), *Oceanospirillales* (24.5%), and *Rhodobacterales* (13.4%) with a marked decrease of SAR11 (5.1%) as dominant taxa (Figure 6). Some differences were seen also for less abundant taxa in BS zone with an increase in *Pseudomonadales* (3.8%), *Desulfobacterales* (1.8%), and *Methylophilales* (1.6%) (Figure 6). In contrast to all the other zones, *Campylobacterales* (2.1%) had a higher relative abundance in all sampling points in the MC zone (Figure 6). Finally, an increase in the number of sequences was observed for the *Sphingomonadales* order at MB (1.9%) and MC zones (Figure 6).

**Figure 7:**
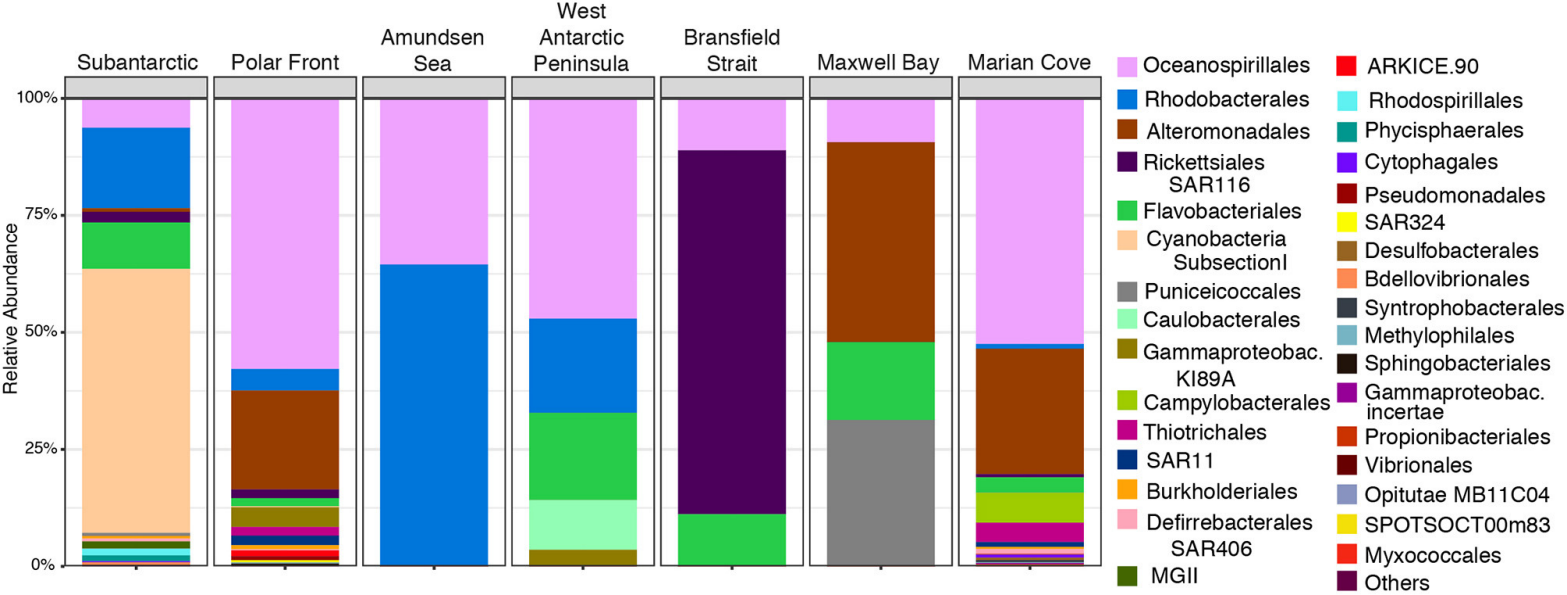
Relative abundance of most abundant indicator OTUs_99_% at the order level in the 16S rRNA-based fraction of each zone in the upper water layer (0, 7, and 10 m combined).

We identified the OTUs that were responsible for the significant difference in community composition observed across the transect with the indicspecies R package. A total of 695 OTUs were selected as the strong indicator species for the 16S rRNA-based data set (*p* = 0.001; stat > 0.82; IV > 0.8) (Supplementary Table 6). The order *Cyanobacteria* characterized the indicator OTUs for the SA, being the most abundant indicator OTU (56%) followed by *Rhodobacterales* (17%) and *Flavobacteriales* OTU (9.8%) (Figure 7 and Supplementary Table 6). However, OTUs of the order *Cyanobacteria* were not restricted to this zone. They were also found in the PF zone, but with a very low percentage of abundance (0.23%) in relation to other indicator OTUs (Supplementary Table 6). The PF zone hosted the highest number of indicators OTUs (316) dominated by members of the *Oceanospirillales* (56.8%) and *Alteromonadales* (20.8%) orders (Figure 7 and Supplementary Table 6). *Rhodobacterales* (4.3%) was the third most dominant order in the PF zone (Supplementary Table 6), and OTUs from the order *Pseudomonadales* were selected as exclusive indicators of this zone (Figure 7 and Supplementary Table 6). AS zone, which had the lowest number of indicators OTUs (Supplementary Table 6), was dominated by *Oceanospirillales* (65%) followed by *Rhodobacterales* (34%) (Figure 7 and Supplementary Table 6).

Indicator OTUs from the order *Caulobacterales* were selected as the exclusive indicator OTU for the WAP zone (Figure 7 and Supplementary Table 6). The order *Rickettsiales* (SAR116) was characterized the BS zone with 77% of the indicator OTUs sequences (Figure 7 and Supplementary Table 6). *Puniceicoccales* was the second major OTUs indicator in the MB zone (31%), being mostly restricted to this zone and the SA zone where it was found in less abundance among the indicators OTUs (0.67%) (Figure 7 and Supplementary Table 6). MC was the second zone that presented a high number of indicators OTUs (281 OTUs). Furthermore, OTUs from the order *Campylobacterales* were selected as exclusive indicator OTUs for this zone (Figure 7 and Supplementary Table 6).

## Discussion

Our study reports the biogeography of marine microbes in a vast and sparsely studied area of the SO. The patterns of community composition show that both the active fraction and the standing stock of bacterioplankton assemblages are structured by both key environmental parameters (temperature, salinity, POC, PON, and silicic acid) (27% of the variance) and by geographical distance (10% of the variance). The estimated amount of variance in our study was similar to the values reported in a review by Hanson et al. (2012). They point out that the average variance explained by environmental effect is 27% compared to 10% for a distance effect, highlighting that the contemporary selection may turn out to have a greater effect on microbial composition than historical processes on the biogeography of microorganisms. Among the factors that could have explained microbial community composition but that we could not measure in this study, the selection, drift, dispersal, and diversification (mutation) are the fundamental ecological processes (Hanson et al., 2012; Nemergut et al., 2016). Dispersal and drift have been identified as the important ecological processes in biogeographical studies conducted in marine environments (Lindström and Langenheder, 2012; Wilkins et al., 2013b).

Spatial distance has been proposed as a barrier for dispersal in marine microorganisms (Cho and Tiedje, 2000; Ramette and Tiedje, 2007). In most of the combinations tested in this study, dissimilarity increased as the geographical distance between communities increased (Figure 5). This strong distance–decay relationship can be associated with decreasing similarity in environmental parameters and hydrography with the increasing geographical distance. However, the amount of variance explained by distance vs. environmental factors will depend on the sampling scale (Martiny et al., 2006). A large spatial geographical distance was covered in our study (~6,500 km) for which distance would be expected to dominate over the environmental variation. However, the environmental characteristics of each sampled zone were stronger in driving the variability of the bacterioplankton communities, pointing out that the observed biogeographical patterns are a combination of both distance and environmental factors.

Strong distance–decay patterns were also encountered in communities that were not geographically distant. This was the case for SA vs. PF zones, AS vs. WAP zones, and BS vs. AS zones, all of them separated by <250 km of distance. Several studies indicate that oceanographic properties of water masses and fronts play a significant role in the structure of bacterioplankton communities (Galand et al., 2010; Wilkins et al., 2013a; Baltar et al., 2016; Djurhuus et al., 2017). The strong shift in community composition observed here within short distances could be due to the presence of different water masses. In particular, some samples obtained within the SA zone were collected in the southern branch of SAF and PF; meanwhile, the majority of the samples from the PF zone were obtained in the sACCf (Supplementary Figure 1). Likewise, the samples collected for the AS zone did not cross any front, unlike those collected in the WAP zone, which mostly established themselves in the sbACC (Supplementary Figure 1). In the case of BS, the samples obtained were located within the sACCf. This result demonstrates that SO fronts can act as significant biogeographical boundaries, generating significant changes in the community composition of spatially near bacterioplankton communities.

**Figure 8:**
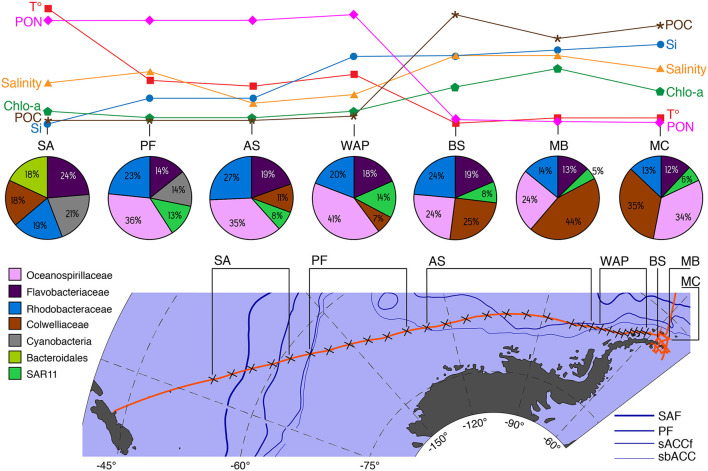
Conceptual description of major taxonomic changes in the upper layer (0, 7, and 10 m) of 16S rRNA-based microbial communities in relation to key physical and chemical environmental parameter and oceanographic features that drive community changes along the spatial transect from Christchurch-New Zealand to South Shetland Islands. Variable abbreviations: T°, temperature °C; PON, particulate organic nitrogen; Salinity, Chlo-a, total chlorophyll; POC, particulate organic carbon; Si, silicic acid. Site abbreviations: SA, Subantarctic; PF, Polar Front; AS, Amundsen Sea; WAP, West Antarctic Peninsula; BS, Bransfield Strait; MB, Maxwell Bay; MC, Marian Cove. Current abbreviations: SAF, Subantarctic Front; PF, Polar Front; sACCf, southern Antarctic Circumpolar Current front; sbACC, southern boundary Antarctic Circumpolar Current.

The bacterioplankton communities were heterogeneously distributed along the SO gradient being structured by the environmental parameters of each zone (Figure 8). According to the environmental parameters, the bacterioplankton communities of the seven zones were divided into two major groups: (i) oceanic areas of the SO transect composed of SA, PF, AS, and WAP zones and (ii) coastal areas of the SO composed of BS, MB, and MC.

The temperature was the main environmental factor that correlated with community composition among the zones possibly restricting the abundance of bacteria such as *Alteromonadales* and SAR11 (Supplementary Table 4) while promoting the appearance of *Cyanobacteria* (Figure 8). In agreement with these findings, it has been shown that the distribution of SAR11 (a globally distributed bacteria) can be significantly affected by temperature changes (Brown et al., 2012). The low temperatures recorded in BS, MB, and MC could act as a growth factor for SAR11 for which high abundances are found at temperatures above 0°C (Brown et al., 2012). Conversely, *Alteromonadales* are cold-adapted heterotrophs that are normally found in cold waters of the SO related to ice melt (Piquet et al., 2011). This may explain their high abundance as indicator taxa in the last part of the transect, a zone associated with ice formation and with the lowest temperatures registered (Figure 8). Furthermore, one of the species that contributed most to the differentiation of the SA community from the rest of the zones was *Cyanobacteria*, which dominated in this zone despite being a minor taxonomic component (0.57%) of the entire data set (Figure 8). The first sample of the SA, where the number of sequences of *Cyanobacteria* was the most abundant, was collected in waters belonging to the southern branch of the SAF (−52.1400°S) (Figure 8). High abundances of *Cyanobacteria* have previously been reported further north of the STF where subtropical waters exhibit higher temperature and salinity (Wilkins et al., 2013a; Liu et al., 2019). Additionally, water surface temperature has been reported as a determining factor in the distribution of *Cyanobacteria* in the SO (Marchant et al., 1987). The water temperature at which the highest abundance of *Cyanobacteria* was found corresponds to 12°C, which represents the highest water temperature recorded in the entire transect, suggesting that the presence of *Cyanobacteria* is related to the water temperature in accordance with the characteristics described for the SAF.

The bacterioplankton communities of the PF, AS, and AP zones were mainly structured by variations in the concentrations of silicic acid, POC, and PON (Figure 8) and second, Chl-a, DON, and ammonium concentrations. The most important nutrients for phytoplankton abundances are nitrogen and phosphorus and, in particular, silicate for diatoms (Sidabutar, xbib2016). However, ammonium has been described to be an important nutrient able to alleviate the photoinhibition or the light-limitation at which phytoplankton is exposed in Antarctic waters (Agustí et al., 2009). Elevated ammonium concentrations (~3.3 μm) were recorded for the AP zone compared to the rest of the transect. Ammonium concentrations are seasonally variable in the WAP with peak concentrations (>3.5 μm) recorded in early autumn (March–May) (Henley et al., 2017). Previous studies have established successional patterns between phytoplankton blooms and some specific bacterioplankton members (Abell and Bowman, 2005; Obernosterer et al., 2011; Kim et al., 2014; Choi, 2016; Luria et al., 2016; Liu et al., 2019). In the PF, AS, and AP zones, the generalist *Oceanospirillales, Rhodobacterales*, and *Flavobacteriales* dominated the indicator taxa. *Flavobacteria, Rhodobacterales*, and *Oceanospirillales* have been positively correlated with the abundance of large and small diatoms in the SO (Kim et al., 2014; Luria et al., 2016; Liu et al., 2019). Considering the environmental parameters and the indicator OTUs selected, it is possible to indicate that the composition of the bacterioplankton community in the AS and AP are tightly related and structured by phytoplankton blooms.

In BS, MB, and MC, the community structure of bacterioplankton was mainly driven by salinity, POC, and silicic acid (Figure 8, Supplementary Figure 4, and Supplementary Table 4). The BS is characterized by a mixture of different bodies of water coming from different sectors of the WAP. The surface layer during winter is characterized by temperatures lower than 1°C and salinity of 34.0 which is defined by Antarctic Surface Water (Hofmann et al., 1996). Meanwhile, MB and MC fjord present a typical estuarine circulation with superficial colder and less saline water (Llanillo et al., 2019). Furthermore, MC registered high concentrations of silicic acid and POC probably associated with meltwater streams with terrigenous particles that persisted until late March (Yoo et al., 2015). The bacterioplankton community composition observed for BS, MB, and MC zones were similar to the composition reported by other studies in the same area, relating the differences in the composition to the environmental gradients rather than spatial distance (Zheng et al., 2011; Moreno-Pino et al., 2016; Kim et al., 2020). In particular, *Campylobacterales* (Epsilonproteobacteria) and *Puniceicoccales* (*Verrucomicrobiales*) were selected as the indicator taxa of MC and MB, respectively. *Campylobacterales* was detected in MC from head to mouth and from 0 to 25 m unlike that reported by Kim et al. (2020), who report a higher abundance of this order in areas near the mouth of the fjord influenced by water from MB. Furthermore, *Verrucomicrobiales* and *Campylobacterales* have been described as the common members of particle-associated communities having the ability to degrade particulate organic carbon sources ((Crespo et al., 2013; Fontanez et al., 2015; Duret et al., 2019)). Differences in the hydrographical properties of the water column, such as melt water input or mixed wind can lead to variation in the microbial community composition of adjacent zones (Zheng et al., 2011; Moreno-Pino et al., 2016). Consequently, differences in the morphological characteristic, sediment load, and meltwater discharge could be responsible for the observed differences in community composition between the zones BS, MB, and MC.

Finally, our results indicate the differences between the total (16S rRNA gene) and active (16S rRNA-based) microbial community composition among the complete data set. The 16S rRNA-based fraction appeared to be more resolutive for describing and explaining the bacterioplankton assemblages of each community compared to the 16S rRNA gene. In addition, in our study, higher α-diversity values were observed for the active community compared to the total community (Figure 4). Furthermore, a higher degree of dissimilarity between the two fractions was observed across the transect (Supplementary Figure 3). The high compositional dissimilarity between the 16S rRNA gene and 16S rRNA-based community may be due to differential changes in the relative abundance of certain OTUs. Traditionally, ribosomal RNA genes are used to identify microorganisms present in a specific environment regardless of their metabolic state, whereas ribosomal RNA has been consistently used to characterize the growth state or activity of microorganisms in cultured and mixed microbial communities (Campbell et al., 2009; Gaidos et al., 2011; Campbell and Kirchman, 2013; Hunt et al., 2013). This is because a positive relationship between the response of bacteria to resource availability and the number of rRNA operons has been shown (Klappenbach et al., 2000), and ribosomal content has been found positively correlated with the growth rate for many bacterial groups (DeLong et al., 1989; Schäfer et al., 2001; Troussellier et al., 2002). The number of 16S rRNA gene operons has been described as the variable in bacteria reflecting different ecological strategies of growth and activity (Moeseneder et al., 2005; Rastogi et al., 2009). However, the number of ribosomes can be even more abundant than rRNA operons depending on the growth rate of the bacteria (Kerkhof and Ward, 1993). Therefore, it could be assumed that in oligotrophic marine environments, such as SO, where bacteria tend to show a slow growth rate and a low number of rRNA operons, some marine bacteria might not be detected at the rRNA gene level but could be still detected at the rRNA level (Fegatella et al., 1998; Moeseneder et al., 2005). This could explain the higher α-diversity observed in the active fraction compared to the total fraction. Furthermore, it has been described that in heterogeneous environments, such as estuaries, environmental variables such as salinity and Chl-a may affect the differential growth rate but not the abundance of individual taxa (Campbell and Kirchman, 2013). Accordingly, we can expect that the bacterioplankton communities in heterogenous gradients will show higher differences between the active and total community in comparison with bacterioplankton communities living in more stable environmental gradients.

## Conclusion

In conclusion, our results show that there were differences in the bacterioplankton community composition across the SO transect from New Zealand to WAP. The differences in taxonomic composition among the seven zones analyzed were strongly correlated with environmental parameters and with geographical distances. The bacterioplankton community turnover was mainly mediated by temperature, silicic acid, PON, salinity, and POC. However, ocean fronts also shaped microbial community composition by increasing physicochemical and biological dissimilarities within restricted geographical areas and thus promoting distinct communities within short distances. Consequently, the biogeographical patterns observed in our study originate from a combination of environmental gradients and oceanographical discontinuities produced by oceanic fronts. In summary, the main contribution of this study is to unveil the complex spatial patterns of the SO marine bacterioplankton communities, at small and large geographical scales (from fjord to ocean), based on the RNA analysis. RNA appeared as a useful tool to explore bacterioplankton community composition across contrasted marine areas. It provided a precise description of complex microbial community dynamics in a changing environment.

## Data Availability Statement

The original contributions presented in the study are included in the article/Supplementary Material. The genomic data presented in the study are available at the European Nucleotide Archive database (ENA) under project identification number PRJEB45020 and sample accession numbers ERS6476988 and ERS6442804.

## Author Contributions

CM-M, PG, and HG wrote the original draft. JI and CM-M collected the microbial diversity samples. JI contributed to review the manuscript. CM-M prepared and processed the 16s rRNA sequencing data. CM-M and PG performed the statistical analysis. S-YH, BL, and I-YA collected and processed the physicochemical water parameters. MC collected the chlorophyll samples and reviewed the manuscript. MV processed the chlorophyl samples and contributed to review the manuscript. CF provided the resources for sampling collection and contributed to review the manuscript. HG and I-YA provided funding and resources for field campaign. All authors have read and agreed to the published version of the manuscript.

## Funding

This research was conducted as a part of the project Carbon cycle change and ecosystem response under the Southern Ocean warming (PE22110) and supported by the Korea Polar Research Institute (KOPRI), Incheon. JI and CM-M were partially supported by the National Agency for Research and Development (ANID) through project FONDAPIDEAL (Grant No. 15150003). CM-M was also financed through Ph.D. ANID Scholarship Doctorado Nacional 2016 (No. 21160201). CF was financed by the National Agency for Research and Development (ANID) through Basal Funding COPAS (FB2100021). MV and MC were funded by the US National Science Foundation–Office of Polar Programs (Award No. 1822289). The data presented are part of the Ph.D. Thesis of CM-M.

## Conflict of Interest

The authors declare that the research was conducted in the absence of any commercial or financial relationships that could be construed as a potential conflict of interest.

## Publisher's Note

All claims expressed in this article are solely those of the authors and do not necessarily represent those of their affiliated organizations, or those of the publisher, the editors and the reviewers. Any product that may be evaluated in this article, or claim that may be made by its manufacturer, is not guaranteed or endorsed by the publisher.
