# Corrigendum: Biogeography of southern ocean active prokaryotic communities over a large spatial scale

**DOI:** 10.3389/fmicb.2023.1224211

**Published:** 2023-06-12

**Authors:** Claudia Maturana-Martínez, José Luis Iriarte, Sun-Yong Ha, Boyeon Lee, In-Young Ahn, Maria Vernet, Mattias Cape, Camila Fernández, Humberto E. González, Pierre E. Galand

**Affiliations:** ^1^Centro de Investigación Dinámica de Ecosistemas Marinos de Altas Latitudes (IDEAL) and Universidad Austral de Chile, Valdivia, Chile; ^2^Sorbonne Université, CNRS, Laboratoire d'Ecogéochimie des Environnements Benthiques, Banyuls-sur-Mer, France; ^3^Division of Polar Ocean Science, Korea Polar Research Institute, Incheon, Republic of Korea; ^4^Scripps Institution of Oceanography, University of California, San Diego, San Diego, CA, United States; ^5^School of Oceanography, University of Washington, Seattle, WA, United States; ^6^Sorbonne Université, CNRS, Laboratoire d'Océanographie Microbienne (LOMIC), Banyuls-sur-Mer, France; ^7^Centro COPAS Coastal, Universidad de Concepción, Concepción, Chile

**Keywords:** DNA, RNA, Southern Ocean, fjord, biogeography

In the published article, there was an error regarding the affiliation for Camila Fernandez. As well as having affiliation [6], she should also have Centro COPAS Coastal, Universidad de Concepción, Concepción, Chile.

In the published article, there was an error in the **Funding** statement. One source of funding was not included. The correct **Funding** statement appears below.

