# Supplementary material for: Biogeography of Southern Ocean Active Prokaryotic Communities Over a Large Spatial Scale
Source: Front Microbiol. 2022 May 3;13:862812. doi: 10.3389/fmicb.2022.862812 (PMC9111744; doi:10.3389/fmicb.2022.862812)
Supplement: Supplementary file 1 [file Data_Sheet_1.docx]

**SUPPLEMENTARY MATERIAL**


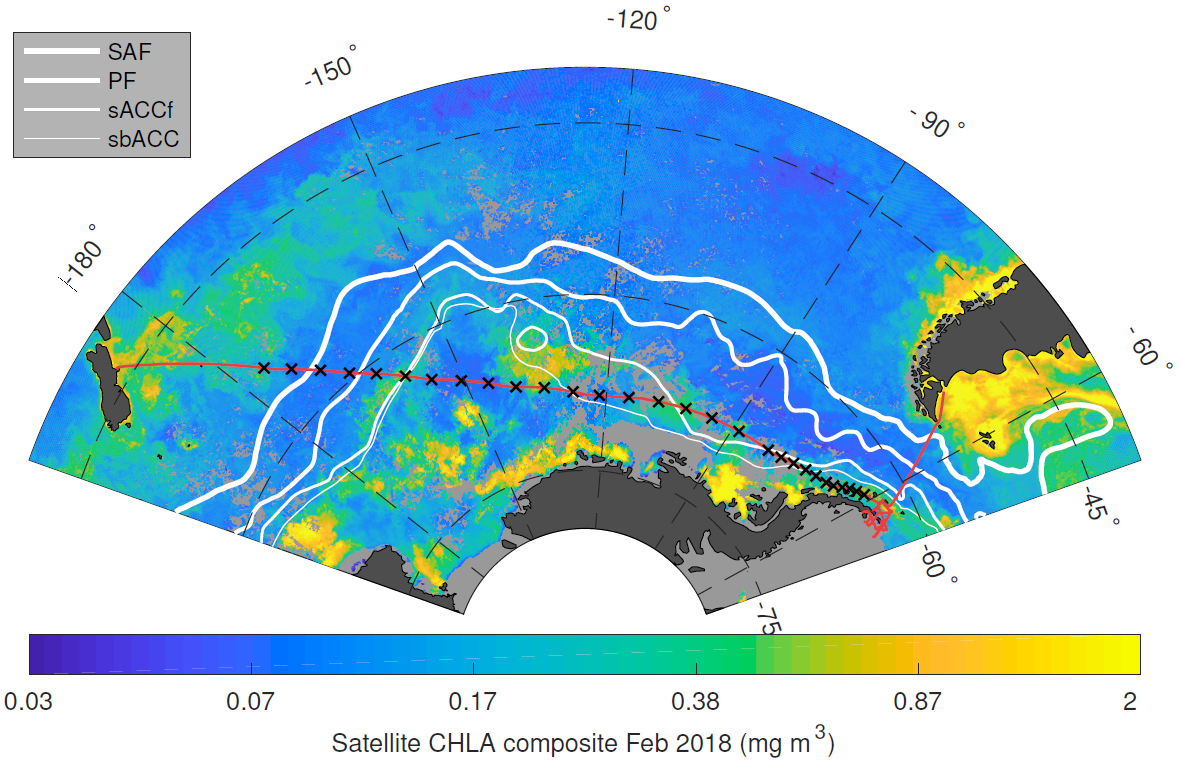


**Supplementary figure 1**: Oceanic fronts of Southern Ocean. SAF, Subantarctic Front; PF, Polar Front; sACCf, Southern Antarctic Circumpolar Current Front; sbACC, southern boundary Antarctic Circumpolar Current. The red line shows the cruise transect and the X denote sampling sites.


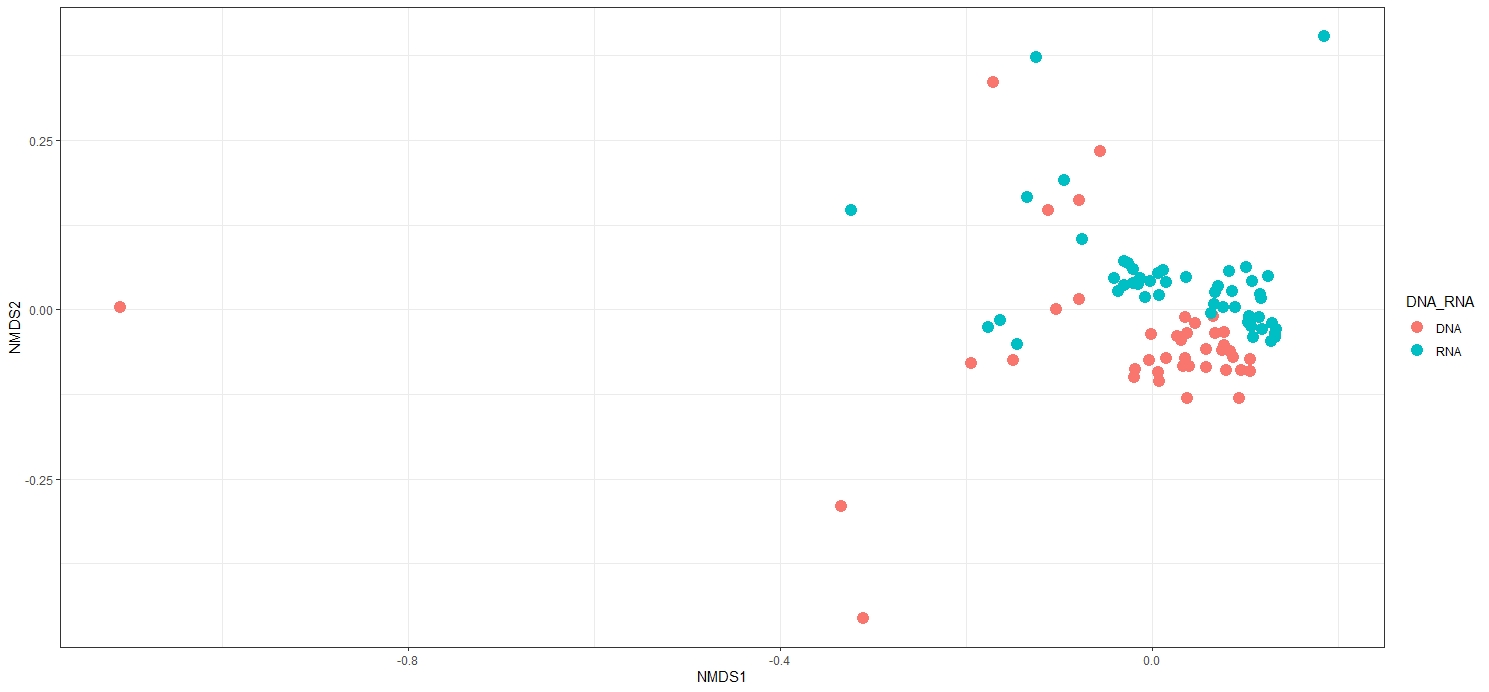


**Supplementary Figure 2**: Non-Metric Multidimensional Scaling ordination (NMDS) based on Bray-Curtis disimilarity of microbial communities identified across the seven zones. Colours represent 16S rRNA gene communities (red) and 16S rRNA-based communities (blue). Stress 0.15.


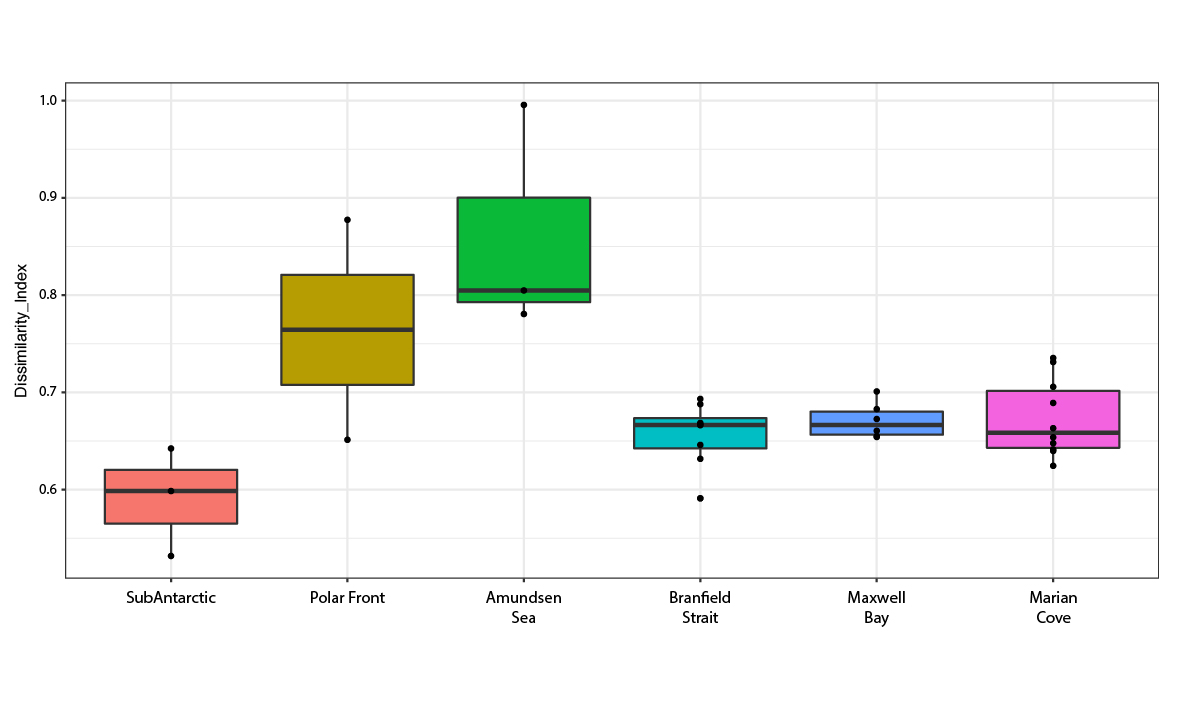


**Supplementary Figure 3**: Bray-Curtis dissimilarity between the 16S rRNA gene and 16S rRNA-based fractions for microbial communities sampled. Only samples for which both the DNA and RNA fractions were successfully amplified were considered for this analysis. Colors represent zones (Subantarctic, Polar Front, Amundsen Sea, Antarctic Peninsula, Bransfield Strait, Maxwell Bay and Marian Cove). The number of samples per zone used is as follows: Subantarctic= 3; Polar Front= 2; Amundsen Sea= 3; Bransfield Strait= 8, Maxwell Bay= 6; Marian Cove= 10.


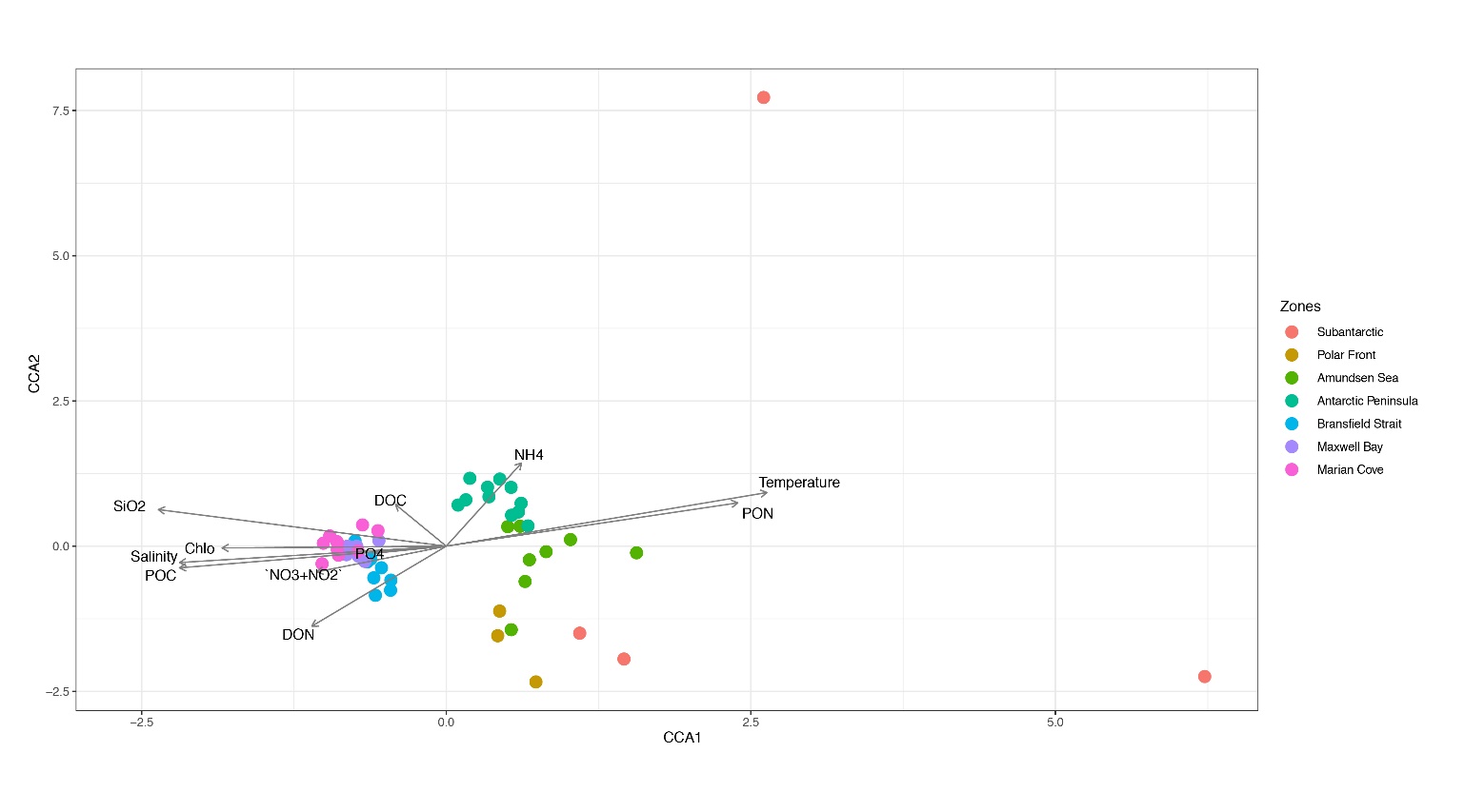


**Supplementary Figure 4:** Partial Canonical Correspondence Analysis (CCA) showing CCA1 and CCA2 plot of environmental and biological (OTU table) variables for the 16S rRNA-based community. Environmental variables correspond to temperature (°C), salinity (PSU), NH_4_= ammonium, NO_2_´+NO_3_= inorganic nitrogen, Chla= total chlorophyll, SiO_2_= silicic Acid, PO_4=_ phosphate, DOC= dissolved organic carbon, POC= particulate organic carbon, DON= dissolved organic nitrogen, PON= particulate organic nitrogen.

**Supplementary Table 1:** Number of sequences detected for bacterial and archaea assemblages in water samples across the Southern Ocean transect.

| **Location** | **Total Reads** | **RNA reads** | **DNA reads** |
| --- | --- | --- | --- |
| Subantarctic | 192679 | 89818 | 102861 |
| Polar Front | 205831 | 126987 | 78844 |
| Amundsen Sea | 327900 | 303128 | 24772 |
| Antarctic Peninsula | 434881 | 434881 | NO DATA |
| Bransfield Strait | 624113 | 261736 | 362377 |
| Maxwell Bay | 510895 | 201339 | 309556 |
| Marian Cove | 889268 | 471733 | 417535 |
| **Total Reads** | 3185567 | 1889622 | 1089468 |

**Supplementary table 2:** Permutational Multivariate Analysis of Variance (PERMANOVA) examining the effects of the variables Depth (0-50 m) and Station (Subantarctic, Polar Front, Amundsen Sea, Antarctic Peninsula, Bransfield Strait, Maxwell Bay and Marian Cove) and 16S rRNA gene/ 16S rRNA-based in all the data set. Key to abbreviations and column headings: D.f, degrees of freedom; MS, mean square; F, F ratio; R^2^, coefficient of determination; P, p-value. Significant results are in bold.

|  | | | | | |
| --- | --- | --- | --- | --- | --- |
| Source of variation | Df | MS | F | R2 | P |
| Zone | 6 | 0.682 | 2.517 | 0.070 | ***0.001*** |
| depth | 3 | 0.264 | 0.974 | 0.009 | 0.498 |
| 16S rRNA gene vs 16S rRNA-based | 1 | 1.028 | 3.790 | 0.035 | ***0.001*** |

**Supplementary table 3**: Partial CCA

| CCA all samples | Inertia | Proportion | CCA1 | % variation | CCA2 | % variation |
| --- | --- | --- | --- | --- | --- | --- |
|  |  |  |  | CCA1 |  | CCA2 |
| Total | 7.1388 | 100% |  |  |  |  |
| Geographic distance | 0.7028 | 10% |  |  |  |  |
| Environmental variables | 1.8948 | 27% | 0.49 | 7.6% | 0.262 | 4% |
| Unconstrained | 4.5412 | 63% |  |  |  |  |

**Supplementary table 4**: partial CCA scores contrasting variables.

| **Variable** | **CCA1** | **CCA2** |
| --- | --- | --- |
| Salinity | **-0.661** | -0.085 |
| Temperature | **0.795** | 0.279 |
| Ammonium | 0.187 | **0.432** |
| Inorganic Nitrogen | -0.321 | -0.134 |
| Chlorophyll a | **-0.555** | -0.010 |
| Silicic Acid | **-0.713** | 0.190 |
| Phosphate | -0.172 | -0.036 |
| Dissolved organic Carbon | -0.126 | 0.217 |
| Particulate Organic Carbon | **-0.661** | -0.113 |
| Dissolved Organic Nitrogen | -0.333 | **-0.416** |
| Particulate Organic Nitrogen | **0.722** | 0.225 |

**Supplementary table** 5: Linear model geographic distance (Km) versus Abundance dissimilarity matrix (Bray-Curtis).

| **Zones combination** | **F-statistic** | **adjusted** | **p-value** |
| --- | --- | --- | --- |
|  |  | **R-squared** |  |
| Subantarctic vs Polar Front | 78.370 | **0.876** | **0.000** |
| Subantarctic vs Amundsen Sea | 6.803 | 0.158 | **0.014** |
| Subantarctic vs Wes Antarctic Peninsula | **138.900** | **0.762** | **0.000** |
| Polar Front vs Amundsen Sea | 14.010 | 0.361 | **0.001** |
| Polar Front vs West Antarctic Peninsula | 37.780 | 0.534 | **0.000** |
| Amundsen Sea vs West Antarctic Peninsula | 163.400 | 0.651 | **0.000** |
| Bransfield Strait vs Subantarctic | **126.200** | **0.802** | **0.000** |
| Bransfield Strait vs Polar Front | 15.230 | 0.382 | **0.001** |
| Bransfield Strait vs Amundsen Sea | 107.100 | 0.628 | **0.000** |
| Bransfield Strait vs West Antarctic Peninsula | 0.369 | -0.007 | 0.545 |
| Bransfield Strait vs Maxwell Bay | 14.750 | 0.226 | **0.000** |
| Bransfield Strait vs Marian Cove | 41.810 | 0.306 | **0.000** |
| Maxwell Bay vs Subantarctic | **121.800** | **0.840** | **0.000** |
| Maxwell Bay vs Polar Front | 18.180 | 0.503 | **0.001** |
| Maxwell Bay vs Amundsen Sea | **141.700** | **0.750** | **0.000** |
| Maxwell Bay vs West Antarctic Peninsula | 0.047 | -0.015 | 0.830 |
| Maxwell Bay vs Marian Cove | 0.173 | -0.014 | 0.680 |
| Marian Cove vs Subantarctic | **292.200** | **0.882** | **0.000** |
| Marian Cove vs Polar Front | 18.850 | 0.381 | **0.000** |
| Marian Cove vs Amundsen Sea | **193.500** | **0.709** | **0.000** |
| Marian Cove vs West Antarctic Peninsula | 0.174 | -0.007 | 0.678 |

**Supplemental table 6:** Distribution of indicator microbial OTU in the 16S rRNA-based communities, among the seven provinces (IV >0.8 p =0.001; stat > 0.8).

|  | **N° Indicator** | **N°** | **Microbial Taxa** | | **Abundance** |
| --- | --- | --- | --- | --- | --- |
| **Province** | **microbial** | **OTUs** |  | | **%** |
|  | **OTUs** | **per Class** | **Class/ Order Level** | | **per Province** |
| Subantarctic | 53 | 10 | Alphaproteobacteria | Rhodobacterales | 17.22 |
|  |  | 4 | Alphaproteobacteria | Rhodospirillales | 1.44 |
|  |  | 4 | Alphaproteobacteria | Rickettsiales SAR116 | 2.3 |
|  |  | 1 | Betaproteobacteria | Burkholderiales | 0.56 |
|  |  | 5 | Cyanobacteria | SubsectionI | 56.43 |
|  |  | 1 | Cytophagia | Cytophagales | 0.27 |
|  |  | 1 | Deferribacteres | Deferribacterales SAR406 | 0.56 |
|  |  | 1 | Deltaproteobacteria | Bdellovibrionales | 0.49 |
|  |  | 1 | Fibrobacteria | Fibrobacterales | 0 |
|  |  | 7 | Flavobacteriia | Flavobacteriales | 9.89 |
|  |  | 3 | Gammaproteobacteria | Alteromonadales | 0.75 |
|  |  | 6 | Gammaproteobacteria | Oceanospirillales | 6.25 |
|  |  | 2 | Gammaproteobacteria | Vibrionales | 0.15 |
|  |  | 1 | Opitutae | MB11C04 | 0.09 |
|  |  | 1 | Opitutae | Puniceicoccales | 0.67 |
|  |  | 2 | Phycisphaerae | Phycisphaerales | 1.22 |
|  |  | 1 | Planctomycetacia | Planctomycetales | 0.08 |
|  |  | 2 | Thermoplasmata | MGII | 1.56 |
| Polar Front | 316 | 29 | Alphaproteobacteria | Rhodobacterales | 4.53 |
|  |  | 6 | Alphaproteobacteria | Rhodospirillales | 1.66 |
|  |  | 13 | Alphaproteobacteria | Rickettsiales SAR116 | 1.8 |
|  |  | 7 | Alphaproteobacteria | SAR11 | 1.98 |
|  |  | 3 | ARKICE-90 | uncultured | 1.24 |
|  |  | 2 | Betaproteobacteria | Burkholderiales | 0.87 |
|  |  | 1 | Betaproteobacteria | Methylophilales | 0.23 |
|  |  | 5 | Cyanobacteria | SubsectionI | 0.23 |
|  |  | 1 | Cytophagia | Cytophagales | 0 |
|  |  | 2 | Deferribacteres | Deferribacterales SAR406 | 0.23 |
|  |  | 1 | Deltaproteobacteria | Bdellovibrionales | 0 |
|  |  | 2 | Deltaproteobacteria | SAR324 | 0.5 |
|  |  | 1 | Fibrobacteria | Fibrobacterales | 0.09 |
|  |  | 14 | Flavobacteriia | Flavobacteriales | 1.71 |
|  |  | 48 | Gammaproteobacteria | Alteromonadales | 20.84 |
|  |  | 5 | Gammaproteobacteria | KI89A | 4.11 |
|  |  | 162 | Gammaproteobacteria | Oceanospirillales | 56.86 |
|  |  | 2 | Gammaproteobacteria | Pseudomonadales | 0.78 |
|  |  | 2 | Gammaproteobacteria | Thiotrichales | 1.84 |
|  |  | 2 | Gammaproteobacteria | Vibrionales | 0 |
|  |  | 1 | Opitutae | MB11C04 | 0.04 |
|  |  | 1 | Opitutae | Puniceicoccales | 0 |
|  |  | 2 | Phycisphaerae | Phycisphaerales | 0 |
|  |  | 1 | Planctomycetacia | Planctomycetales | 0 |
|  |  | 1 | Sphingobacteriia | Sphingobacteriales | 0.41 |
|  |  | 2 | Thermoplasmata | MGII | 0 |
| Amundsen Sea | 2 | 1 | Alphaproteobacteria | Rhodobacterales | 65.51 |
|  |  | 1 | Gammaproteobacteria | Oceanospirillales | 35.48 |
| West Antarctic Peninsula | 23 | 1 | Alphaproteobacteria | Caulobacterales | 10.63 |
|  |  | 6 | Alphaproteobacteria | Rhodobacterales | 20.15 |
|  |  | 3 | Flavobacteriia | Flavobacteriales | 18.64 |
|  |  | 1 | Gammaproteobacteria | KI89A | 3.5 |
|  |  | 12 | Gammaproteobacteria | Oceanospirillales | 47.05 |
| Bransfield Strait | 3 | 1 | Alphaproteobacteria | Rickettsiales | 77.77 |
|  |  | 1 | Flavobacteriia | Flavobacteriales | 11.11 |
|  |  | 1 | Gammaproteobacteria | Oceanospirillales | 11.11 |
| Maxwell Bay | 17 | 3 | Flavobacteriia | Flavobacteriales | 16.66 |
|  |  | 8 | Gammaproteobacteria | Alteromonadales | 42.71 |
|  |  | 2 | Gammaproteobacteria | Oceanospirillales | 9.375 |
|  |  | 4 | Opitutae | Puniceicoccales | 31.25 |
| Marian Cove | 281 | 1 | Actinobacteria | Propionibacteriales | 0.15 |
|  |  | 7 | Alphaproteobacteria | Rhodobacterales | 1.04 |
|  |  | 3 | Alphaproteobacteria | Rickettsiales SAR116 | 0.67 |
|  |  | 3 | Alphaproteobacteria | SAR11 | 1.02 |
|  |  | 1 | ARKICE-90 | uncultured | 0.19 |
|  |  | 3 | Betaproteobacteria | Burkholderiales | 0.46 |
|  |  | 2 | Betaproteobacteria | Methylophilales | 0.19 |
|  |  | 2 | Cytophagia | Cytophagales | 0.65 |
|  |  | 4 | Deferribacteres | Deferribacterales 406 | 1.02 |
|  |  | 1 | Deltaproteobacteria | Desulfobacterales | 0.53 |
|  |  | 1 | Deltaproteobacteria | Myxococcales | 0.12 |
|  |  | 1 | Deltaproteobacteria | Syntrophobacterales | 0.48 |
|  |  | 25 | Epsilonproteobacteria | Campylobacterales | 6.39 |
|  |  | 10 | Flavobacteriia | Flavobacteriales | 3.3 |
|  |  | 74 | Gammaproteobacteria | Alteromonadales | 26.8 |
|  |  | 1 | Gammaproteobacteria | Incertae | 0.24 |
|  |  | 126 | Gammaproteobacteria | Oceanospirillales | 52.47 |
|  |  | 15 | Gammaproteobacteria | Thiotrichales | 4.13 |
|  |  | 1 | SPOTSOCT00m83 |  | 0.13 |

**Supplementary Table**: geographic location of the main transect and geographic location of the zone divisions (sampling stations).

| Global Transect | Latitud | Longitud |
| --- | --- | --- |
| Christchurch NZ | 52° 14.371 120 S | 178° 4.22 W |
| West Antarctic Peninsula | 63° 23.711’ S | 61° 18.164’ W |
| Bransfield Strait | 62° 50.3990 S | 58° 12.0028 W |
| Marian Cove | 62° 12.8495 S | 58° 46.3919 W |
| Zones (sampling stations) |  |  |
| Subantarctic zone (SA) | 52° 14.2651 S | 59° 28.0279 S |
| Polar Front (PF) zone | 60° 57.023 S | 66° 12.1361 S |
| Amundsen Sea (AS) zone | 67° 9.2437 S | 67° 26.722 S |
| Antarctic Peninsula (WAP) zone | 67° 22.1291 S | 63° 24.9352 S |
| Bransfield Strait (BS) zone | 62° 50.3983 S | 62° 28.8008 S |
| Maxwell Bay (MB) zone | 62° 17.1021 S | 62° 13.8023 S |
| Marian Cove (MC) zone | 62° 13.0785’S | 62° 12.2998 S |


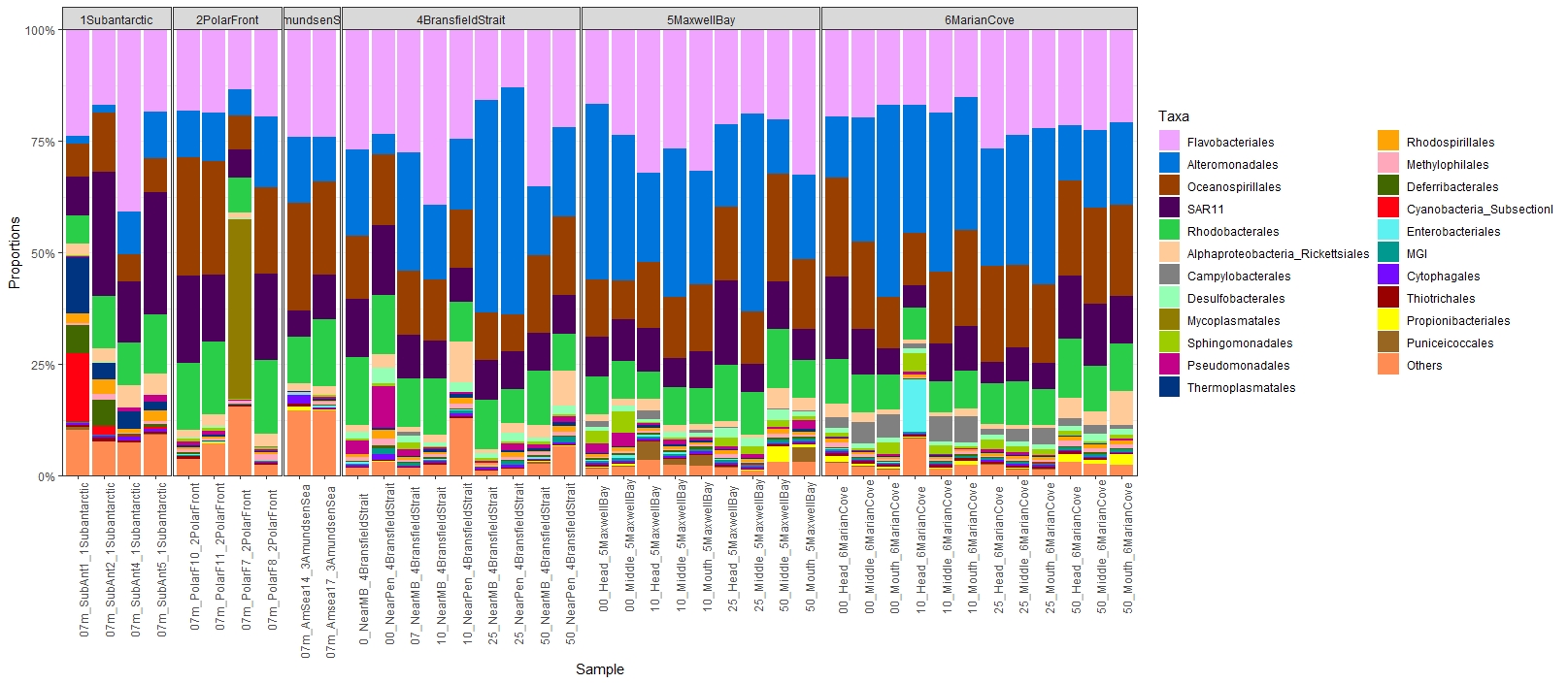


**Supplementary figure 5:** Relative abundance of taxonomical groups at the Order level in the 16S rRNA gene fraction of each zone in the depths sampled (0, 7, 10, 25 and 50 m).
